# CNTools: A computational toolbox for cellular neighborhood analysis from multiplexed images

**DOI:** 10.1371/journal.pcbi.1012344

**Published:** 2024-08-28

**Authors:** Yicheng Tao, Fan Feng, Xin Luo, Conrad V. Reihsmann, Alexander L. Hopkirk, Jean-Philippe Cartailler, Marcela Brissova, Stephen C. J. Parker, Diane C. Saunders, Jie Liu

**Affiliations:** 1 Department of Electrical Engineering and Computer Science, University of Michigan, Ann Arbor, Michigan, United States of America; 2 Department of Computational Medicine and Bioinformatics, University of Michigan, Ann Arbor, Michigan, United States of America; 3 Department of Medicine, Vanderbilt University Medical Center, Nashville, Tennessee, United States of America; 4 Center for Stem Cell Biology, Vanderbilt University, Nashville, Tennessee, United States of America; European Molecular Biology Laboratory, UNITED KINGDOM OF GREAT BRITAIN AND NORTHERN IRELAND

## Abstract

Recent studies show that cellular neighborhoods play an important role in evolving biological events such as cancer and diabetes. Therefore, it is critical to accurately and efficiently identify cellular neighborhoods from spatially-resolved single-cell transcriptomic data or single-cell resolution tissue imaging data. In this work, we develop CNTools, a computational toolbox for end-to-end cellular neighborhood analysis on annotated cell images, comprising both the identification and analysis steps. It includes state-of-the-art cellular neighborhood identification methods and post-identification smoothing techniques, with our newly proposed Cellular Neighbor Embedding (CNE) method and Naive Smoothing technique, as well as several established downstream analysis approaches. We applied CNTools on three real-world CODEX datasets and evaluated identification methods with smoothing techniques quantitatively and qualitatively. It shows that CNE with Naive Smoothing overall outperformed other methods and revealed more convincing biological insights. We also provided suggestions on how to choose proper identification methods and smoothing techniques according to input data.

## Author summary

Cellular neighborhoods (CNs), defined as cell regions with similar cell type composition, are attracting more and more attention because of their unique influence on biological processes in many diseases. However, a reliable method that can identify biologically meaningful CNs under different data settings is missing. Therefore, we provide such a method named Cellular Neighbor Embedding (CNE) with Naive Smoothing, which overall outperforms state-of-the-art methods on three real-world datasets. In addition, we make an easy-to-use toolbox that supports multiple CN identification pipelines and various downstream analyses, which can help researchers compare CN results and pursue more biological insights form CNs.

## Introduction

Powered by evolving multiplexed tissue imaging technologies like CODEX [1], the roles of cellular microenvironments in biological tissues have been identified in many pathological events such as cancer and diabetes [2–8]. Cellular neighborhoods (CNs), which reflect special cellular microenvironments, are commonly defined as cellular regions of the tissue with homogeneous local cell type (CT) compositions [5, 8]. Studies showed that between different groups of patients, the CT-CN relationships as well as CN-CN relationships are differential and associated with the characteristics of the patient groups, such as survival time [5, 8]. These findings shed any light on the importance of characterizing and analyzing CNs as biologically-meaningful cellular microenvironments. On the other hand, cellular microenvironments can be defined in forms other than CNs. Kim et al. [9] regards cellular microenvironments as clusters of cells with similar local aggregated marker intensities. Wu et al. [10] builds cellular microenvironments based on graph neural network embeddings trained on cell expression profiles and patient-level properties. Javed et al. [11] constructs cellular microenvironments out of cell image patches with similar CT connection frequencies. None of these works promise to produce microenvironments with similar local CT compositions, making them beyond the scope of this work.

**Fig 1:**
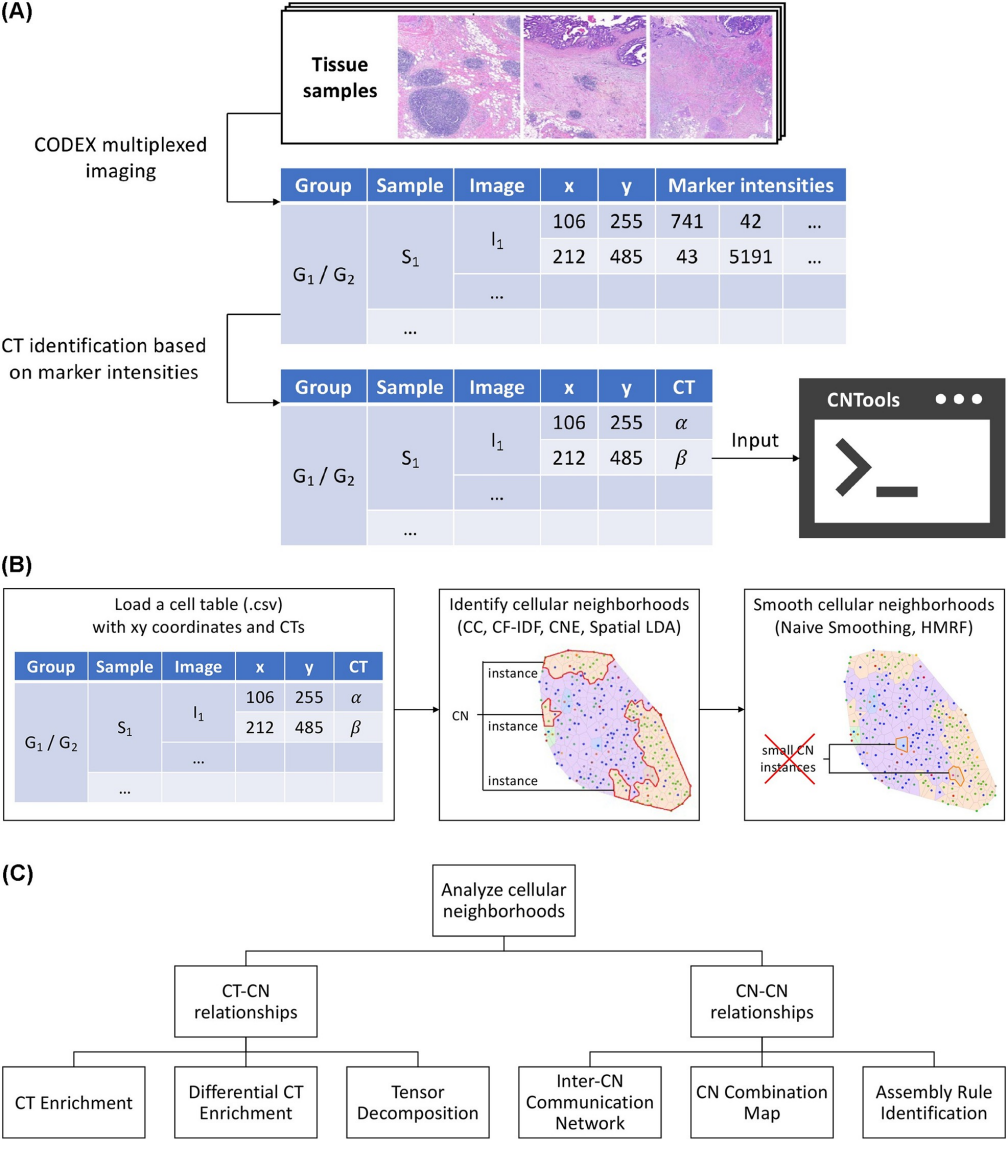
Overview of CNTools. (A) The input of CNTools can be generated from raw tissues using CODEX and CT identification from marker intensities. (B) CNTools follows the workflow of loading cells and identifying, smoothing, and analyzing CNs. (C) Downstream CN analysis in CNTools reveals CT-CN and CN-CN relationships.

Accurate CN characterization is crucial to reveal new biological insights in downstream spatial analysis [3, 5–8]. As a result, several CN identification methods on annotated cell images have recently been proposed. Schürch et al. [5] uses a *k*-means method focusing on local CT compositions, termed CC, that represents each cell by its nearby CT frequencies and then clusters cells into CNs, which is straightforward but ignores the facts that neighboring cells may have different importances based on their distances to the target cell and CT frequency can be imbalanced in the data [5, 8], hindering the identification of CNs corresponding to less frequent CTs. Dynamic CF-IDF [6], termed CF-IDF, detects cell communities in an inverse distance-weighted cell graph and represents each community by its CT frequency normalized by overall CT frequency, similar to TF-IDF [12], and finally clusters communities into CNs using *k*-means. CF-IDF handles the CT frequency imbalance problem, but cell communities are found only using cell locations, which may not be able to reflect spatial CT distributions. Spatial LDA [7] relies on latent Dirichlet allocation [13, LDA] with spatial regulations to assign CN labels to cells without clustering. Specifically, it regards each cell’s CT as a “word”, each cell and its neighbors as a “document”, and each CN type as a “topic” in LDA, and introduces a prior that neighbors are more likely to have similar CN preferences. In another direction, the CN identification task is similar to the problem of inductive community detection on attributed graphs in the machine learning field, with a distance-based cell graph. Thus, two state-of-the-art methods ClusterNet [14] and GAP [15] were considered in experiments. Both of them follow the architecture of using graph neural networks to embed nodes and leveraging metrics for community detection such as modularity [16] and normalized cut [17] as the loss function. A common problem of these methods is that they may produce small CN instances that are less biological meaningful (Figs 1B and A, B, H, and I in S1 Text, left columns). CN instances are defined as connected components in CNs given a graph constructed by cell-cell distances, such as Delaunay triangulation graph [18] and *k*-NN graph. How to post-process small CN instances, the process we name as CN smoothing, is another challenge for accurate CN characterization in addition to CN identification.

To overcome the drawbacks of CN identification method CC and handle CN smoothing, we proposed a new CN identification method named Cellular Neighbor Embedding (CNE), inspired by *t*-S(tochastic)N(eighbor)E(mbedding) [19]. Alongside, we provided two CN smoothing techniques, (i) newly proposed Naive Smoothing that removes small CN instances based on similarity of local cell representations and (ii) HMRF [20] that builds hidden Markov random field models on cell graphs with CTs as observances and CN types as hidden states.

Another challenge for accurate CN characterization is how to evaluate CNs, since ground truth is usually unavailable [5, 8]. As far as we know, CN identification methods with smoothing techniques have not yet been systematically compared. We handled this challenge in two folds. Quantitatively, we introduced Shannon entropy of CT conditioned on CN and mean CN instance size as metrics measuring “purity” and “granularity”, respectively. Qualitatively, we used six established downstream CN analysis methods studying CT-CN or CN-CN relationships to see whether main conclusions of the original studies could be revealed with the same data. Meanwhile, since it is unlikely that a single method can work best in all situations, researchers may need a collective implementation of CN tools that can help them conveniently identify CNs in different settings, benchmark their performance, and compare their analysis results. Hence, we implemented CNTools, a computational toolbox that includes four CN identification methods (CC, CF-IDF, Spatial LDA, and our CNE), two CN smoothing techniques (Naive Smoothing and HMRF), and six established downstream CN analysis methods studying CT-CN or CN-CN relationships. The identified CNs from different CN identification methods with smoothing techniques were evaluated on three CODEX datasets from disparate human tissues, both quantitatively and qualitatively. Overall, CNE with Naive Smoothing outperformed other methods and revealed more convincing biological insights, which validates its effectiveness in detecting neighborhood structures among cells. Suggestions on how to choose CN identification methods and smoothing techniques given different input image sizes are also provided at the end of the paper. With CNTools, researchers can easily and accurately identify CNs and pursue biological insights from CNs.

## Results

### Overview of CNTools

The entire workflow of CNTools comprises four consecutive steps, (i) cell loading, (ii) CN identification, (iii) CN smoothing, and (iv) CN analysis. First, it takes as input a cell table in CSV format with the sample group ID, sample ID, image ID, xy coordinates, and CT as attributes, which can be generated from raw tissues using CODEX and CT identification from marker intensities (Fig 1A and 1B). Additional information such as marker positivities may be required by downstream analyses. Second, it identifies CNs given a user-specified number of CNs, i.e., gives each cell a label representing a unique CN, using one of the four CN identification methods, CC, CF-IDF, CNE, and Spatial LDA (Fig 1B). Third, it smooths the identified CNs, i.e., re-assigns each cell a CN label to improve smoothness of CN distributions, using one of the two CN smoothing techniques, Naive Smoothing and HMRF (Fig 1B). Fourth, it analyzes CNs focusing on CT-CN relationships via CT Enrichment [5], Differential CT Enrichment [5], and Tensor Decomposition [5], and CN-CN relationships via Inter-CN Communication Network [5], CN Combination Map [8], and Assembly Rule Identification [8] (Fig 1C). Details about various methods in CNTools are provided in the Methods section.

CNTools includes two new approaches, a CN identification method named CNE and a CN smoothing method named Naive Smoothing. CNE introduces three modifications to CC. First, it uses perplexity measurement to assign different weights to neighboring cells based on distances similar to *t*-SNE. Specifically, it represents each cell *x* as a vector whose *i*-th entry is the sum of the probability densities of the cells belonging to CT *i* under a spatial Gaussian centered at *x*. The variance of the Gaussian is adapted based on the *t*-SNE’s perplexity measurement, i.e., entropy of the neighboring cells’ Gaussian densities. Second, it normalizes cell representations by a similar technique inspired by CF-IDF. Each representation is *ℓ*_1_-normalized to get local distance-weighted CT frequencies and then element-wise multiplied by the log inverse overall CT frequencies to alleviate the CT frequency imbalance problem. Third, in order to improve CN smoothness, it includes a spatial regularizer in *k*-means algorithm that encourages similar cluster representations among neighboring cells during clustering cell representations. Finally, Naive Smoothing was proposed to post-process CNs for smoothness, which uses edges in Delaunay triangulation of cell images to define neighbors and utilizes cell representations to re-assign each cell in small CN instances to the CN of its neighbor that resides in a large CN instance and has the most similar representation.

### Performance comparison of CN identification methods

**Fig 2:**
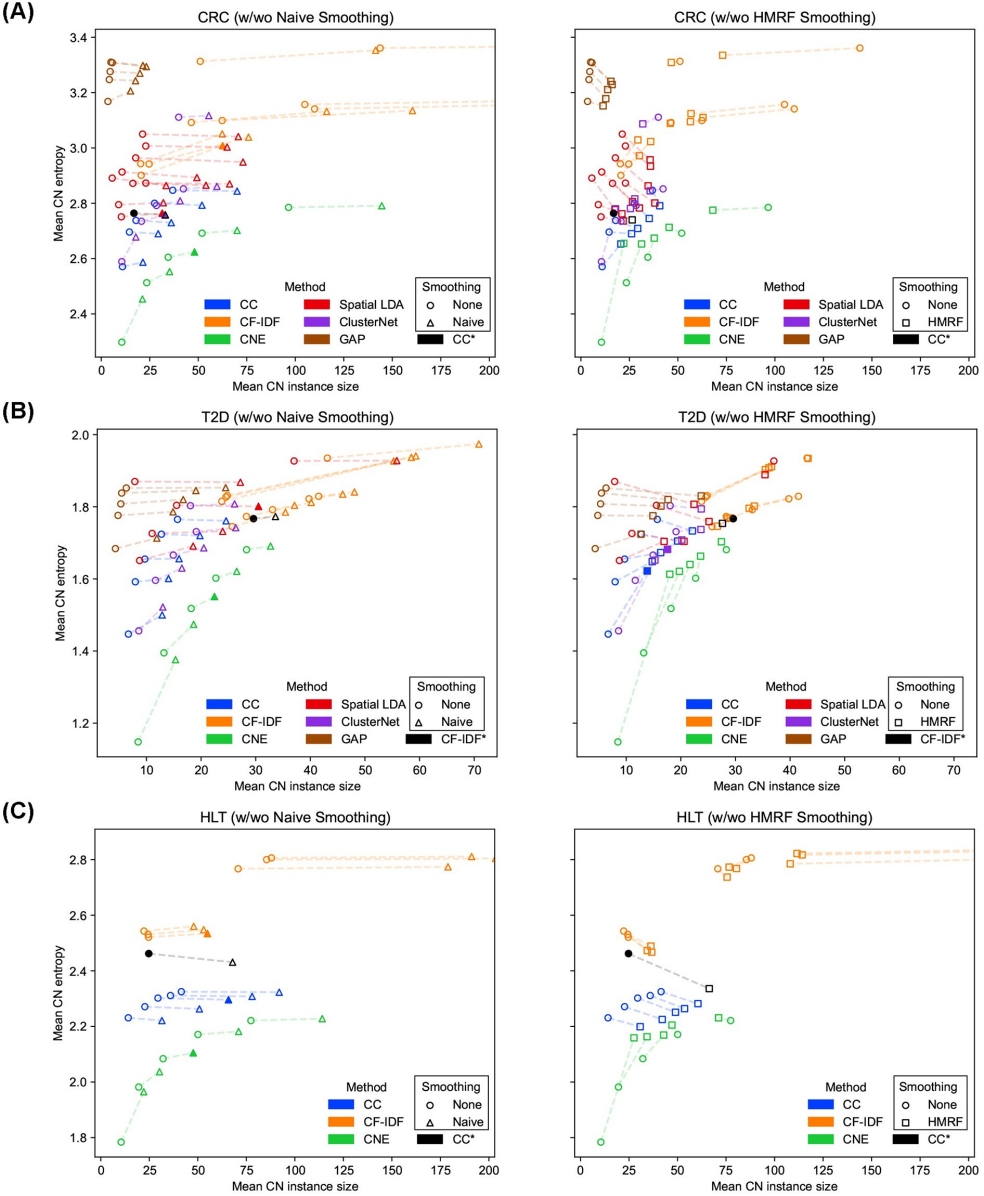
Performance comparison of CN identification methods. (A)–(C) The performance of CN identification methods on the CRC, T2D, and HLT datasets, respectively. Each data point represents a CN result. The shape of data point is decided by how it has been smoothed. The dashed lines connect CN results under different smoothing techniques. The *x* axis shows the granularity of CNs and larger *x* means lower granularity. The *y* axis shows the purity of CNs and larger *y* means lower purity. Filled markers represent CNs with selected hyperparameters. The results used by the original studies are marked with asterisks. CNE consistently produced purer CNs over other methods under various granularities on different datasets.

Accurate CNs are the prerequisite for successful downstream analysis. To evaluate CN identification methods with smoothing techniques in CNTools quantitatively, we utilized three public CODEX datasets, colorectal cancer [5, CRC], type 2 diabetes [6, T2D], and human lymphoid tissues [8, HLT], which have all been previously used for CN identification and analysis. We adapted the original published pipelines to pre-process data to make sure all methods could run in a common setting for comparison (Experiment settings of CN identification, Methods), and selected hyperparameters in a reasonable scope for all methods according to whether they could reveal similar CNs as the original results and had a biologically meaningful CN visualization (Figs 2A and A–C in S1 Text). Though we did not manually choose the number of the CNs, in reality that number can be determined by visualizing CNs and examining CT Enrichment analysis results so that CNs are biologically meaningful and non-redundant [5, 7, 8], or through clustering heuristics such as Silhouette Coefficient [21] and Gap Statistic [22].

Methods were benchmarked by two metrics, namely purity and granularity of CNs. The purity of CNs was assessed through the Shannon entropy of CT conditioned on CN, short as *mean CN entropy* (Metric to evaluate CN purity, Methods). A lower entropy value leads to higher purity, which means that each CN is mostly made up of one or few CTs. In contrast, higher entropy or lower purity means that CNs have more evenly distributed CTs. The granularity of CNs was assessed through *mean CN instance size*, the average number of cells in each CN instance defined by Delaunay triangulation. A larger size number gives lower granularity, which means cells of the same CN are more likely to be neighbors in opposite to being separated by cells of other CNs. Generally speaking, higher granularity comes with higher purity and vice versa. When mean CN instance size goes to its minimum 1, i.e., each cell makes up of its own CN, mean CN entropy becomes 0 and purity reaches its highest. With the growth of mean CN instance size, neighboring cells with similar local CT distribution form CN instances, however, these cells are not necessarily of the same CTs, and the larger CN instances are, the more likely they contain multiple CTs and represent a more even CT distribution, which increases mean CN entropy and degrades purity. When mean CN instance size goes to its maximum, i.e., all cells belong to one CN, mean CN entropy becomes the entropy of all cells and purity goes to its lowest, which should be considered as the worst case of a CN identification method. To avoid trivial CN results, we should consider granularity in a reasonable range for each dataset, and since there is no gold granularity, several levels of granularity should be tried. On the other hand, CNs with high purity are more biological meaningful as a cellular microenvironment and easier to analyze with a clearer biological definition than impure CNs. Therefore, a better CN identification method should identify CNs of higher purity under various granularities in a reasonable range. To compare different methods, we varied their hyperparameters to identify CNs of different granularities and then compared their CN purity trends.

The CRC dataset includes tissue images from 35 advanced-stage colorectal cancer patients, of which seventeen exhibit Crohn’s-like reaction (CLR) with longer survival and eighteen present diffuse inflammatory infiltration (DII) with shorter survivals. It has an average number of 1,846 cells in each image, and the cells have been annotated into 28 CTs. In terms of CN identification (Fig 2A), our CNE method consistently produced purer CNs compared with other methods under different granularities, which is desirable. Meanwhile, Spatial LDA produced CNs with higher purity and higher granularity, whereas CF-IDF identified CNs with lower purity and lower granularity, which made it difficult to compare Spatial LDA and CF-IDF quantitatively. ClusterNet produced better CNs than CF-IDF and Spatial LDA, though there is an outlying result. GAP performed much worse compared to all other methods. For CN smoothing (Fig 2A), both Naive Smoothing and HMRF decreased the granularity of raw CNs in most cases, which demonstrated their smoothing effectiveness. On the other hand, Naive Smoothing basically kept the purity and lowered the granularity of raw CNs identified by each method, while HMRF increased purity for impure CNs and decreased purity for pure CNs. Naive Smoothing better kept the purity-granularity trends of raw CNs, while HMRF tended to break these trends by transferring raw CNs into a small regime in the metric space, which indicated that Naive Smoothing is more interpretable than HMRF in smoothing. By visualizing raw and smoothed CNs, we also observed that HMRF modified raw CNs in a less predictable way (Figs A and B in S1 Text).

The T2D dataset includes islet images from six non-diabetic (ND) and ten type-2-diabetic (T2D) donors. Each donor has one large image, which contains 43 islets on average. Each islet is given a unique image ID and has an average number of 97 cells, and the cells have been annotated into ten CTs. In terms of CN identification (Fig 2B), our CNE method outperformed other methods. CC and Spatial LDA produced comparable results, and ClusterNet produced slightly better results than them, which were left to qualitative comparison in downstream analyses. GAP still performed much worse compared to all other methods. For CN smoothing (Fig 2B), similar findings were obtained as on the CRC dataset (Figs H and I in S1 Text).

The HLT dataset includes images of four human lymphoid tissues including two tonsils, a spleen, and a lymph node. It has an average number of 421,516 cells in each image, and the cells have been annotated into 24 CTs (excluding “ECM” cells not relevant to our analysis). In terms of CN identification (Fig 2C), our CNE method outperformed other methods, while CF-IDF produced much less purer CNs than other methods, indicating its challenge with larger images. This might be because in larger images, cells usually do not appear in clear communities and CNs should be identified more based on spatial CT distributions, which made it difficult for CF-IDF to use graph algorithm to find communities which can form pure CNs (Fig A panel B in S1 Text). For CN smoothing (Fig 2C), similar findings were obtained as on the CRC and T2D datasets (Fig O in S1 Text).

Based on these quantitative comparison and visualization of raw and smoothed CNs, hyperparameters and smoothing techniques were chosen for each identification method on each dataset (Tables A–C in S1 Text). In the following sections, we conducted qualitative evaluation of CNs through downstream analysis. We compared different methods to see whether they could reveal the original findings of the CRC [5], T2D [6], and HLT [8] datasets. Since GAP was not performing well, we excluded it from further CN analysis.

### Colorectal cancer affects the functionality of T cells in multiple CNs and stimulates immune processes related to T cell and macrophage enriched CNs

**Fig 3:**
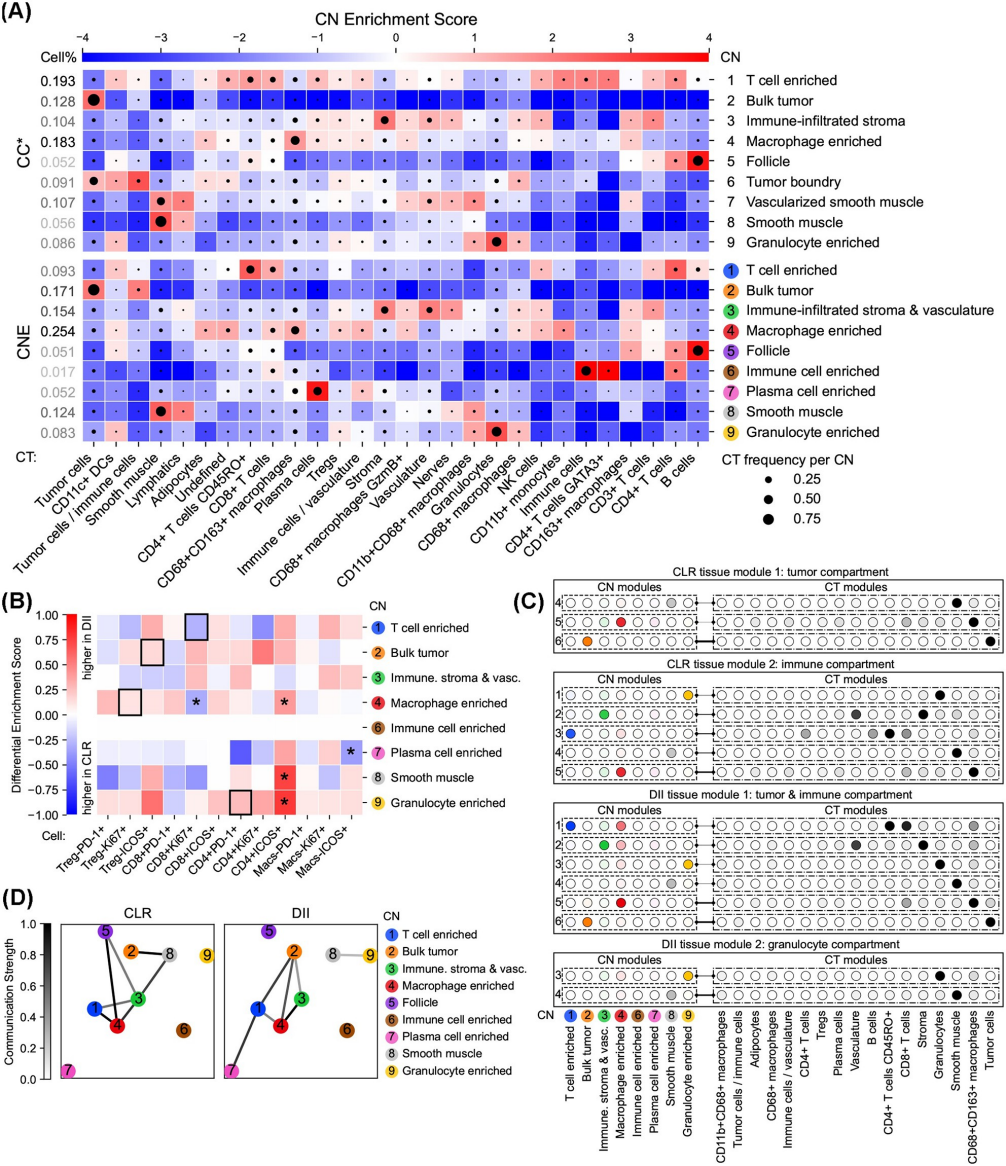
Colorectal cancer affects the functionality of T cells in multiple CNs and stimulates immune processes related to T cell and macrophage enriched CNs. (A) CT Enrichment analysis for CNE and the original CC results (CC*) on the CRC dataset. CNE produced purer CNs enriched by one CT or several related CTs than other methods (Figs C–F panel A in S1 Text). Enrichment scores are given by CT-CN position weight matrices (PWMs). High enrichment scores are colored by red, showing high enrichment of CTs in CNs. CTs are annotated in the original dataset, and CNs are generated by CNTools for each of the three methods indicated and named as the most enriched CTs. CT frequencies per CN are represented by the sizes of black circles in the corresponding blocks. Cell frequencies per CN are listed in the leftmost column. (B) Differential CT Enrichment analysis for CNE on the CRC dataset. CNE identified Ki-67+CD8+ T cells more enriched in the T cell enriched CN in CLR donors, Ki-67+ Treg cells more enriched in the macrophage enriched CN in DII donors, ICOS+ Treg cells more enriched in the bulk tumor CN in DII donors (black boxes in rows 1, 4, and 2), and PD-1+CD4+ T cells more enriched in the granulocyte enriched CN in DII donors (black box in row 9). Differential enrichment scores are given by the coefficients of donor group variables in linear models estimating CT frequencies per CN from overall CT frequencies (*p < 0.05). A CT is more enriched in a CN among DII/CLR donors when the corresponding differential enrichment score is further from zero (closer to 1/-1). (C) Tensor Decomposition analysis for CNE on the CRC dataset. CNE had a tumor compartment and an immune compartment as tissue modules in CLR donors, and a tumor & immune compartment and a granulocyte compartment as tissue modules in DII donors. CNE had a CN module with high weights for T cell and macrophage enriched CNs, whose corresponding CT module had high weights for T cells and macrophages, only in DII donors (DII tissue module 1 row 1). The CT-CN-donor tensor in each donor group is decomposed by non-negative Tucker tensor decomposition. The transparency of circles and lines indicates CT or CN weights in modules and interaction strengths between CT and CN modules, respectively. Only modules with interaction strengths > 0.1 are shown. (D) Inter-CN Communication Network analysis involving {PD1+, Ki-67+, ICOS+}CD8+ T cells and Ki-67+ Tregs for CNE on the CRC dataset. CNE found the follicle CN connected to immune CNs only in CLR donors and that the tumor CN had a stronger connection to the macrophage enriched CN in DII donors. Each node represents a particular CN according to the number on it. The communication strength between each CN pair is determined by the [0, 1]-normalized significance (> 0.9) of the largest canonical correlation in CCA considering involved CTs.

With identified CNs from the CRC dataset, CT Enrichment analysis was first performed to interrogate each CN (Figs 3A and C–F panel A in S1 Text). For CNE, each CN was enriched by one CT (e.g., granulocytes) or several related CTs (e.g., T cells of different functional states), which showed its high purity and biological interpretability. In addition, each CN corresponded to a CN in the original study [5] with similar CT enrichments except the impure “tumor boundry” CN, demonstrating that CNE is capable of identifying important CNs. By contrast, such a CN correspondence could not always been found for the CNs identified by other methods, since some of them were enriched by multiple irrelevant CTs (e.g., CN-5 of CF-IDF and CN-7 of Spatial LDA).

To further investigate the influence of donor groups on CT enrichments in CNs, Differential CT Enrichment analysis was conducted using T cells in different functional states (Figs 3B and C–F panel B in S1 Text). The CN enriched by B cells was removed for CNE and Spatial LDA following the original study, which was not available for CF-IDF. (i) CNE identified Ki-67+CD8+ T cells more enriched in the T cell enriched CN in CLR donors, Ki-67+ Treg cells more enriched in the macrophage enriched CN in DII donors, and ICOS+ Treg cells more enriched in the bulk tumor CN in DII donors (Fig 3B, black boxes in rows 1, 4, and 2), showing that immunosuppressive activity is increased in macrophage enriched and bulk tumor CNs in DII donors, while in CLR donors cytotoxic activity is increased in the T cell enriched CN. On the other hand, CF-IDF and ClusterNet failed to find ICOS+ Treg’s enrichment, and Spatial LDA failed to explicitly find Ki-67+ Treg cell’s enrichment since two macrophage enriched CNs had disparate results. (ii) All tested methods found PD-1+CD4+ T cells more enriched in the granulocyte enriched CN in DII donors, showing its potential contribution to the antitumoral response (Fig 3B, black box in row 9). To sum up, CNE and CC could best support the original findings.

In addition, to discover under each donor group the main factors in CT and CN spaces and how they interact with each other, Tensor Decomposition was applied (Figs 3C and C–F panel C in S1 Text). (i) All methods had a tumor compartment and an immune compartment as tissue modules in CLR donors, and a tumor & immune compartment and a granulocyte compartment as tissue modules in DII donors. (ii) All methods except CF-IDF had a CN module with high weights for T cell and macrophage enriched CNs, whose corresponding CT module had high weights for T cells and macrophages, only in DII donors (Fig 3C, DII tissue module 1 row 1). Based on these findings, all methods except CF-IDF could come to the original conclusion that tumors in DII donors are more correlated to the immune processes with increased coupling between T cell and macrophage enriched CNs.

On the CN-CN interaction side, Inter-CN Communication Network was built to quantify the communication strengths between CNs involving {PD1+, Ki-67+, ICOS+}CD8+ T cells and Ki-67+ Treg cells (Figs 3D and C–F panel D in S1 Text). (i) CNE and CC found the follicle CN connected to immune CNs only in CLR donors, indicating that the processes occurring in the follicle could play a role in the immune activity related to functional T cells. (ii) Only Spatial LDA found the granulocyte enriched CN connected to the tumor CN only in DII donors, congruent with the second finding of Differential CT Enrichment analysis. (iii) All methods except CF-IDF found that the tumor CN had a stronger connection to the macrophage enriched CN in DII donors, showing that the communication of functional T cells between the tumor and the macrophage enriched CN has been increased in DII donors. Furthermore, CNE and Spatial LDA also found DII-exclusive connection between tumor and T cell enriched CNs, which emphasized the results from Differential CT enrichment analysis. However, no methods could support the original conclusions that T cell and macrophage enriched CNs could communicate in functional T cells with the bulk tumor via the tumor boundary, and the communication between tumor boundary and bulk tumor CNs could be disrupted in DII donors. We suspect that this may be because no methods could produce a well-identified tumor boundary CN as in the original study. Further discussion about why this would happen appears in the Discussion section.

### Type 2 diabetes depletes endothelial cells and pericytes in the beta cell enriched CN and promotes CN communication involving vascular and immune cells

**Fig 4:**
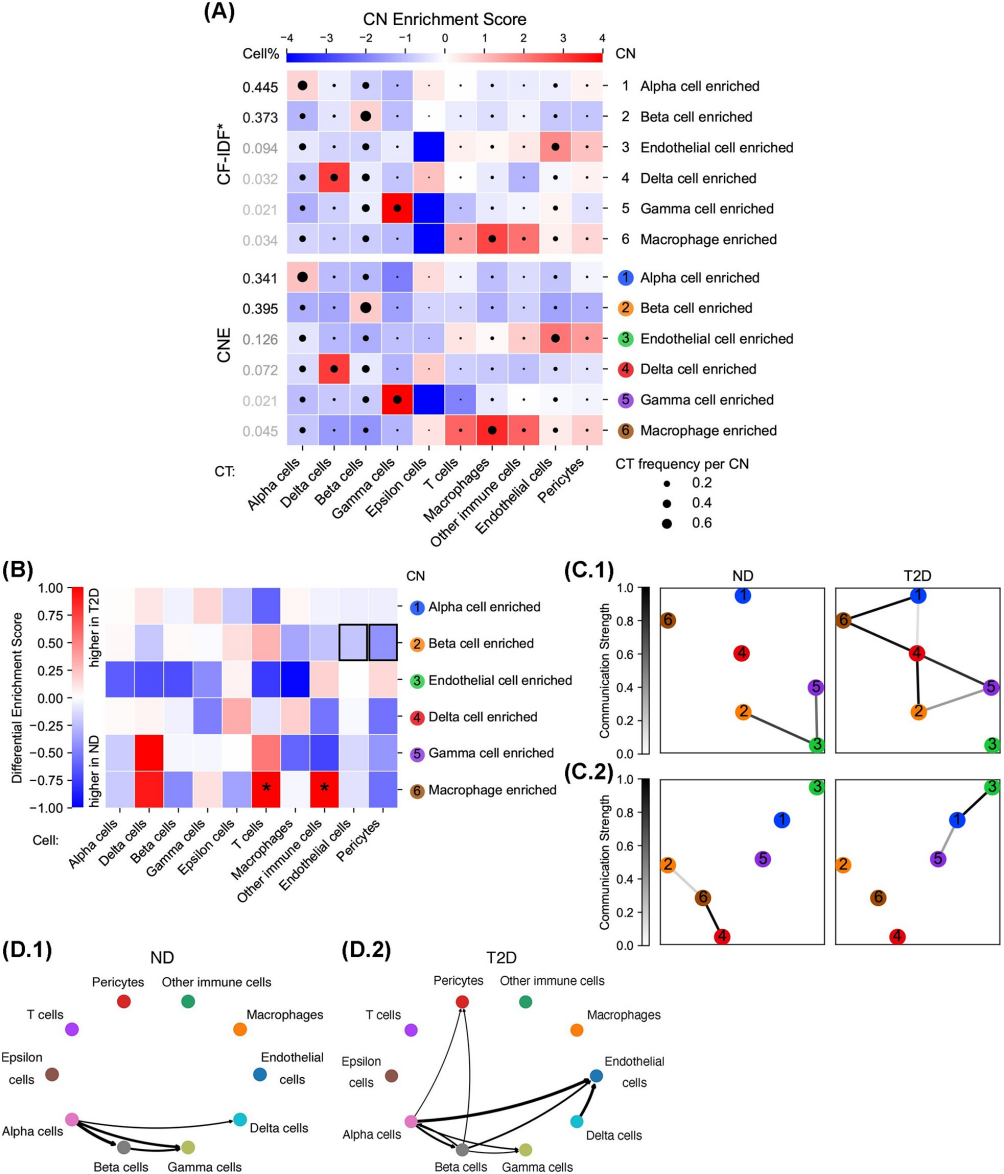
Type 2 diabetes depletes endothelial cells and pericytes in the beta cell enriched CN and promotes CN communication involving vascular and immune cells. (A) CT Enrichment analysis for CNE and the original CF-IDF results (CF-IDF*) on the T2D dataset. CNE produced purer CNs enriched by one CT or several related CTs than other methods. Enrichment scores are given by CT-CN PWMs. CTs are annotated in the original dataset, and CNs are generated by CNTools for each of the three methods indicated and named as the most enriched CTs. CT frequencies per CN are represented by the sizes of black circles in the corresponding blocks. (B) Differential CT Enrichment analysis for CNE on the T2D dataset. CNE found endothelial cells and pericytes less enriched in the beta cell enriched CN in T2D donors (black boxes). Differential enrichment scores are given by the coefficients of donor group variables in linear models estimating CT frequencies per CN from overall CT frequencies (*p < 0.05). A CT is more enriched in a CN among T2D/ND donors when the corresponding differential enrichment score is further from zero (closer to 1/-1). The biological meaning of each CN is provided in (A). (C) Inter-CN Communication Network analysis involving (C.1) vascular cells (endothelial cells and pericytes) or (C.2) immune cells (T cells, macrophages, and other immune cells) for CNE on the T2D dataset. CNE found more communication involving vascular cells between CNs in T2D donors, and more communication involving immune cells between alpha and endothelial cell enriched CNs. Each node represents a particular CN according to the number on it. The communication strength between each CN pair is determined by the [0, 1]-normalized significance (> 0.9) of the largest canonical correlation in CCA with involved CTs. The biological meaning of each CN is provided in (A). (D) CT-CT communication analysis on the T2D dataset. CNE showed a T2D-specific increased communication between alpha and endothelial cell enriched CNs. Communication strengths from sender to receiver CTs are denoted by the darkness and thickness of the corresponding directed lines.

With identified CNs from the T2D dataset, CT Enrichment analysis was first performed to interrogate each CN (Figs 4A and J–M panel A in S1 Text). For CNE, each CN was enriched by one CT (e.g., beta cells) or several related CTs (e.g., endothelial cells and pericytes), which showed its high purity and biological interpretability. In addition, each CN corresponded to a CN in the original study with similar CT enrichments, demonstrating that CNE is capable of identifying important CNs. By contrast, such a CN correspondence could not always been found for the CNs identified by other methods, since some of them were enriched by multiple irrelevant CTs (e.g., CN-4 of CC and CN-6 of Spatial LDA).

Differential CT Enrichment analysis was conducted using all CTs (Figs 4B and J–M panel B in S1 Text). All tested methods except Spatial LDA found endothelial cells and pericytes less enriched in the beta cell enriched CN in T2D donors (Fig 4B, black boxes in row 2), congruent with the original finding of increasing distance between beta and endothelial cells in T2D donors.

Inter-CN Communication Network analysis was carried on using vascular cells (endothelial cells and pericytes) or immune cells (T cells, macrophages, and other immune cells) (Figs 4C and J–M panel C in S1 Text). All tested methods found more communication involving vascular cells between CNs in T2D donors, validating the original finding of upregulated EC-specific signals in T2D donors. Additionally, we noticed an interesting T2D-specific increased immune cell communication between alpha and endothelial cell enriched CNs, given by CNE and the original result. This finding was also validated by CT-CT interaction results. NCEM [23] was employed to calculate CT-CT communication strengths, which showed more active communication between alpha and endothelial cells existing in T2D donors (Fig 4D). Gene network analysis in the original study using bulk RNA-seq of purified alpha cells demonstrated exclusive alpha-endothelial cell communication in T2D donors through VEGF-VEGFR2 and ROBO/SLIT receptor signaling. This unknown immune-related interaction between alpha and endothelial cells in T2D donors may deserve more attention in future research.

### In all human lymphoid tissues, local interactions involving the T cell enriched CN necessitate the presence of B cells, while the light zone CN is not always surrounded by the B cell enriched CN

**Fig 5:**
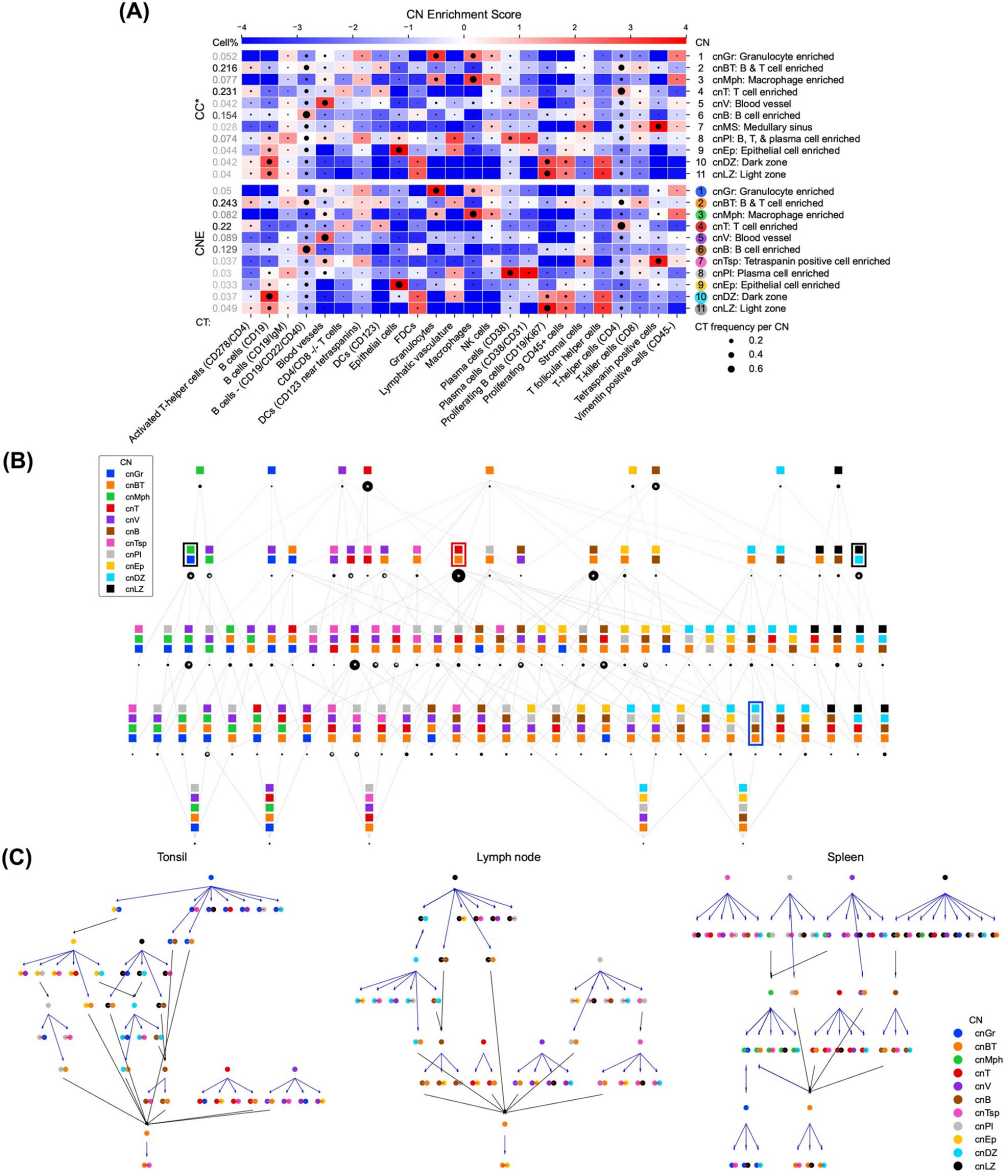
In all human lymphoid tissues, local interactions involved the T cell enriched CN necessitate the presence of B cells, while the light zone CN is not always surrounded by the B cell enriched CN. (A) CT Enrichment analysis for CNE and the original CC results (CC*) on the HLT dataset. CNE produced purer CNs enriched by one CT or several related CTs than other methods. Enrichment scores are given by CT-CN PWMs. CTs are annotated in the original dataset, and CNs are generated by CNTools for each of the three methods indicated and named as the most enriched CTs. CT frequencies per CN are represented by the sizes of black circles in the corresponding blocks. (B) CN Combination Map analysis for CNE on the HLT dataset. Most findings of CNE matches the original study [8], which is better than CC and CF-IDF mainly because of the purity of CNs. (i) cnLZ and cnDZ, and cnGr and cnMph, were more likely to be restricted together than separated (black boxes). (ii) cnV and cnBT existed in SCs combined with many other CNs. The wide spread of cnV agrees with the biological fact that blood vessels are ubiquitous in biological activities. (iii) cnB and cnT had a large SC just containing themselves and cnBT had a small one. No methods found cnPI alone in a SC, but CNE and CF-IDF could support its abundance among SCs. (iv) cnT occurred much more often with cnBT in SCs than without it, suggesting that local interactions between cnT and other CNs necessitate the presence of B cells (red boxes). (v) the outer zones [24], SCs made up of cnB, cnBT/cnT, and cnLZ/cnDZ, were found by all three methods, while only CNE gave such a SC with cnPI, showing a niche within the follicle for plasma cells [25] (blue box). A cell is assigned to a SC, set of CNs, if more than 90% of its nearest 100 cells are assigned to one of those CNs and if it is the minimal set of CNs that has this property. The number of cells in each SC is represented by the size of the black circle below it. The top 20 largest SCs are marked with asterisks. (C) Assembly Rule Identification analysis for CNE on the HLT dataset. CNE preserved more conserved assembly rules (cnT → cnBT-cnT, cnV → cnV-cnPl, cnLZ → cnB-cnLZ, cnB → cnB-cnPl, and cnB → cnB-cnBT) than CC, which in turn reproduced more than CF-IDF. Furthermore, in the spleen, CNE produced cnLZ → cnLZ-cnB and cnLZ → cnLZnotX for other existing CNs, showing that cnLZ was surrounded only by cnB, while in the tonsil and lymph nodes, cnLZ → cnBT-cnLZ was produced, indicating that cnLZ was surrounded not only by cnB. Each node represents a motif, where the edge between CNs is gray if CNs are adjacent and red otherwise. Each edge represents an assembly rule, whose color is blue if it extends 70% of the source motif instances to the target motif or black if the source motif is part of the target motif. Two tonsil images were merged.

With identified CNs from the HLT dataset, CT Enrichment analysis was first performed to interrogate each CN (Figs 5A and P and Q panel A in S1 Text). For CNE, each CN was enriched by one CT except cnBT, which also existed in the original study, showing high CN purity and biological interpretability. In addition, each CN corresponded to a CN in the original study with similar CT enrichments, except cnTsp enriched by tretraspanin positive cells not originally defined, demonstrating that CNE is capable of identifying important CNs. By contrast, such a CN correspondence could not always been found for the CNs identified by other methods due to impure CNs (e.g., cnVT of CC and cnLZDZ of CF-IDF).

CN Combination Map was constructed to detect spatial combinations of CNs, named as spatial contexts (SCs), such that a cell is assigned to a SC if most of its neighbors belong to a CN in the SC and such combination is minimal (Figs 5B and P and Q panel B in S1 Text). SCs are biologically important since interactions between CNs might be reflected in biological processes occurring in such regions [8]. Most findings of CNE matched the original study, better than CC and CF-IDF mainly because of the purity of CNs, though the results related to cnPI were not consistent, probably caused by the enrichment of B cells and T cells in the original cnPI. (i) For CNE and CC, the CN pairs of cnLZ and cnDZ, and cnGr and cnMph, were more likely to be restricted together than separated (Fig 5B, black boxes), while CF-IDF just directly gave cnLZDZ and cnGrMph. (ii) For CNE and CC, cnV and cnBT existed in SCs combined with many other CNs, while CF-IDF also verified cnV but not cnBT, as it only had separated cnB and cnT. The wide spread of cnV agreed with the biological fact that blood vessels are ubiquitous in biological activities. (iii) For CNE and CC, cnB and cnT had a large SC just containing themselves and cnBT had a small one, while CF-IDF could only verify cnB and cnT. No methods found cnPI alone in a SC, but CNE and CF-IDF could support its abundance among SCs. (iv) For CNE, cnT occurred much more often with cnBT in SCs than without it (Fig 5B, red box), suggesting that local interactions between cnT and other CNs necessitate the presence of B cells. CF-IDF provided a similar finding with cnBT replaced by cnB, while the finding was less obvious in the result of CC due to the existence of a large SC composed of cnT and cnVT. (v) The outer zones [24], SCs made up of cnB, cnBT/cnT, and cnLZ/cnDZ, were found by CC, CNE and CF-IDF, while only CNE gave such a SC with cnPI (Fig 5B, blue box), showing a niche within the follicle for plasma cells [25].

In addition, Assembly Rule Identification was performed to detect causal relationships between CNs (Figs 5C and P and Q panel C in S1 Text), aiming to find propagation of biological signals from one CN to the other. In this analysis, a motif is defined as a graph with CNs as nodes connected by two kinds of edges: (i) cnX-cnY, which indicates an instance of cnX is adjacent to an instance of cnY, and (ii) cnXnotY, which indicates an instance of cnX is not adjacent to any instance of cnY. The assembly rules are extracted by extending the motif node-by-node, e.g., cnX → cnX-cnY, which means that given cnX’s instances, most of them are adjacent to cnY’s instances. We considered the rules between one-CN and two-CN motifs as the original study. Conserved assembly rules among all images were first studied, which were (i) cnT → cnBT-cnT, (ii) cnV → cnV-cnPl, (iii) cnLZ → cnB-cnLZ, (iv) cnB → cnB-cnPl, and (v) cnB → cnB-cnBT. Note that cnPl was a highly-mixed CN enriched by B, T, and plasma cells which could have similar effects as cnBT in forming assembly rules. For CNE, (i), (iii), and (v) were directly identified, and (ii) and (iv) were potentially identified by cnV → cnBT-cnV and (v), respectively. Additionally, cnB → cnLZ-cnB was not identified in any tissue. This together with (iii) and (v) suggested that signals propagating from cnLZ to cnB-cnBT could reflect the essential role of cnLZ in lymphoid tissues. For CC, (i) and (v) were directly identified, (ii) and (iv) were potentially identified by cnVT → cnBT-cnVT and (v), receptively, and (iii) was not identified. For CF-IDF, (i), (ii), and (iii) were not identified, and (iv) and (v) were both potentially identified by cnB → cnT-cnB. Second, assembly rules unique to each image were investigated. For CNE and CC, in the spleen, cnLZ → cnLZ-cnB and cnLZ → cnLZnotX for other existing CNs were produced, showing that cnLZ is surrounded only by cnB, while in the tonsil and lymph nodes, cnLZ → cnBT (cnPI originally)-cnLZ was also produced, indicating that cnLZ is surrounded not only by cnB. CF-IDF however could not support these findings due to the lack of cnLZ. In brief, CNE preserved more original conserved assembly rules than CC, which in turn preserved more than CF-IDF, and (iv) was very likely covered by (v).

### Computational complexity

We further investigate the time and space complexity of CN identification methods and smoothing techniques in CNTools. Let n=|C|, d=|T|, k=|N|, and *m* denote the average number of neighbors considered for each cell, where T denotes the set of all CTs and N denotes the set of all CN types. Recall that clustering cells using *k*-means with *i* iterations costs *O*(*kdni*) time and *O*(*n*) space. CC is bottlenecked by its cell representation step both in time and space, which leads to *O*(*mn* log *n* + *kdni*) and *O*((*d* + *m*)*n*) complexity, respectively. CF-IDF is bottlenecked by the Louvain algorithm in time and its community representation step in space, which lead to *O*(*mn* + *kdni*) and $O(d\tilde{n})$ complexity for $\tilde{n}$ communities, respectively. CNE originally is bottlenecked by its cell representation step in both time and space, which leads to *O*(*dn*^2^ + *kdni*) and *O*(*n*^2^) complexity, respectively. However, these complexities can be reduced to *O*(*mn* log *n* + *kdni*) and *O*((*d* + *m*)*n*), the same as CC, by considering only *m* nearest neighbors instead of all other cells when calculating Gaussian densities for each cell and letting remaining densities be zeros. Spatial LDA does not perform clustering and costs *O*(*dmni*′) time for running *i*′ iterations and *O*((*d* + *m*)*n*) space. In reality, Spatial LDA usually costs much more time than the other three methods (Table 1). Naive Smoothing costs $O(dm\hat{n})$ time for $\hat{n}$ cells that need smoothing and *O*(1) space. HMRF costs *O*(*mn* log *n* + *kni*″) time for running *i*″ iterations and *O*((*k* + *m*)*n*) space. It is clear that Naive Smoothing runs much faster than HMRF (Table 1).

**Table 1:** Running time of CN identification methods with different smoothing techniques on the HLT dataset.

Running time (s)		CC	CF-IDF	CNE	Spatial LDA	ClusterNet	GAP
Identification		44.83	323.97	354.42	> two days	OOM	OOM
Smoothing	Naive	224.34	296.25	180.67	-	-	-
	HMRF	1559.72	1151.43	1588.96	-	-	-

## Discussion

In summary, we proposed a computational toolbox, CNTools, for cellular neighborhood identification and analysis. We validated the effectiveness of all tools we implemented in CNTools through extensive experiments on real-world datasets, especially our newly proposed CNE with Naive Smoothing. We believe CNTools is a convenient toolbox for researchers who want to interrogate cellular neighborhoods in single-cell resolution tissue imaging data and pursue new biological insights from them.

The quantitative and qualitative evaluation experiments in our study comprehensively compared all CN identification methods coupled with smoothing techniques in CNTools. In general, CNE with Naive Smoothing revealed more convincing biological insights than other methods in our experiments. We credit this to two facts. First, CNE produced purer CNs, which reduced ambiguity in downstream analysis. Second, CNE could vary the granularity of CNs conveniently by tuning one hyperparameter in a relatively small range and worked well with specific hyperparameter values in all experiments, while other methods either had more hyperparameters to tune (CF-IDF and Spatial LDA) or had to search in a wider range for a good hyperparameter value (CC). However, we also point out that with some careful data pre-processing or post-processing using expertise, other methods, such as CC on the CRC dataset and CF-IDF on the T2D dataset, could achieve even more biologically meaningful results than CNE. This indicates that different methods should be tried to ensure the validity of findings and that additional data manipulation beyond CNTools could improve CN identification results. When it comes to other methods, we found that CF-IDF did not performed well on medium or large-scale data both quantitatively and qualitatively, which might be because it does not take spatial CT distributions into considerations when using graph algorithm to find communities, as aforementioned. There is no clear qualitative performance gap between CC, Spatial LDA, and ClusterNet, however, we always found it easier to have a clear and decisive qualitative conclusion when CNs are pure, which we believe is the main reason why they lagged behind CNE. On large data, methods that rely heavily on iterative graph optimization, i.e., Spatial LDA, ClusterNet, and GAP, run very slow or failed due to the scalability problem of the graph algorithm [26]. On the other hand, we found that Naive Smoothing often produced more reasonable results than HMRF since the latter is less interpretable in smoothing raw CNs (Figs A and B in S1 Text), however, when some of the raw CNs are impure, using HMRF could probably increase purity, such as CC and ClusterNet results on the T2D dataset.

Finally, we checked the hyperparameter sensitivity of CNE with Naive Smoothing by varying CNE’s *perp* ∈ {10, 12.5, 15, 17.5, 20} on the CRC dataset. Quantitatively, we computed the Normalized Mutual Information (NMI) scores, a metric widely used to determine the quality of clustering [27], of all CNE results with various parameters. The NMI score ranges from 0 to 1 and a higher score indicates a greater degree of similarity between two clusters. We took *perp* = 15 as the ground truth and got NMI scores [0.805, 0.785, 1.000, 0.777, 0.771], showing that CNE is robust to *perp* varying from a wide range. Qualitatively, Figs R–V in S1 Text showed that the CN analysis results were not sensitive to hyperparameter changes, and similar biological insights could be obtained from these results.

Nevertheless, CNE also has the problem of producing overly pure CNs that may fail to capture the complexity of the tissues. In the Inter-CN Communication Network analysis on the CRC dataset, the original conclusion could not be reproduced because no methods could produce a well-identified tumor boundary CN as in the original study. By further investigating the CT frequency of this CN, we found that the original tumor boundary CN is highly mixed, whose entropy is 3.31 and top 5 major cell types are tumor cells (39.36%), CD68+CD163+ macrophages (9.78%), granulocytes (7.70%), stroma (6.66%), and tumor cells / immune cells (4.45%). CNE is more capable of detecting pure CNs, which explains why it fails to identify this CN. But, as we have mentioned, different methods may miss key biological insights on different datasets, which stimulates our goal of building CNTools to enable investigators to choose from multiple methods to maximize biologically meaningful output.

With these points in mind, we herein provide several suggestions for users on how to choose CN identification methods and smoothing techniques. First, always start with CNE or CC for CN identification because they are faster and adapt to images of all sizes. CNE is more preferable because it is not sensitive to hyperparameters and provides purer CNs. Second, when image sizes are large (∼10^5^ cells), be cautious when using CF-IDF about impure CNs and try to use ROIs or subsets of the images for Spatial LDA, ClusterNet, and GAP for an efficient run. Third, when raw CNs are already pure or ideal, use Naive Smoothing. Otherwise, try HMRF to increase purity or remove biological ambiguity in CNs.

## Methods

In this section, for any cell *x* we denote its CT by *t*_*x*_, its CN type by *n*_*x*_, its neighbors by *G*_*x*_, and the CN instance containing *x* by *N*_*x*_. Other notations of frequently-used mathematical concepts are listed in Table 2.

**Table 2:** Notations of frequently-used mathematical concepts.

Notation	Mathematical concept
*F*(*t*)	Overall frequency of CT *t*
*F*^*d*^(*t*)	Frequency of CT *t* in donor *d*
$F_{n}^{d}\left(t\right)$	Frequency of CT *t* in donor *d* and CN *n*
C	Set of all cells
$C_{n}$	Set of cells in CN *n*
$C_{n,t}$	Set of cells in CN *n* of CT *t*
T	Set of all CTs
N	Set of all CN types

### Metric to evaluate CN purity

The Shannon entropy, widely used in information theory, is defined as

$$\begin{array}{c} H\left(X\right)=-\sum_{x\in X}P\left(X=x\right)\cdot {\text{log}}_{2}\;P\left(X=x\right). \end{array}$$ (1)

for random variable *X*. We used the Shannon entropy of CT conditioned on CN to evaluate the purity of CNs, which could be written as

$$\begin{array}{c} \begin{array}{cc} H(CT|CN) & =\sum_{n\in N,t\in T}P\left(CN=n,CT=t\right)\cdot {\text{log}}_{2}\frac{P(CN=n)}{P(CN=n,CT=t)}\\ & =\sum_{n\in N,t\in T}\frac{\left|C_{n,t}\right|}{\left|C\right|}\;{\text{log}}_{2}\;\frac{\left|C_{n}\right|}{\left|C_{n,t}\right|}, \end{array} \end{array}$$ (2)

where C, $C_{n}$, and $C_{n,t}$ denote the set of all cells, cells in CN *n*, and cells in CN *n* of CT *t*, respectively (Tables 2 and 3).

**Table 3:** Important abbreviations and terms.

Category	Abbreviation/Term	Explanation
General	CT	Cell type
	CN	Cellular neighborhood, a cellular region of the tissue with a homogeneous local CT composition
	CN instance	A connected component in a CN given a graph constructed by cell-cell distances, such as Delaunay triangulation graph [18] and *k*-NN graph
Evaluation	Purity of CN	Mean CN entropy, the Shannon entropy of CT conditioned on CN
	Granularity of CN	Mean CN instance size, the average number of cells in each CN instance defined by Delaunay triangulation
Dataset	CRC	Annotated CODEX images of tissues from 35 advanced-stage colorectal cancer patients, of which seventeen exhibit Crohn’s-like reaction (CLR) with longer survival and eighteen present diffuse inflammatory infiltration (DII) with shorter survivals
	T2D	Annotated CODEX images of islets from six non-diabetic (ND) and ten type-2-diabetic (T2D) donors
	HLT	Annotated CODEX images of four human lymphoid tissues including two tonsils, a spleen, and a lymph node
Identification & smoothing	CC	A CN identification method based on local CT compositions and *k*-means clustering
	CF-IDF	A CN identification method based on “CT frequency–inverse dataset frequency” representations of cell communities detected by the Louvain algorithm and *k*-means clustering
	CNE	A CN identification method based on distance-weighted CT compositions using Gaussian densities and spatially-regularized *k*-means clustering
	Spatial LDA	A CN identification method based on latent Dirichlet allocation with spatial regulations which regards each CT as a “word”, each neighborhood as a “document”, and each CN type as a “topic”, and introduces a prior on CN preferences of all cells such that neighbors are more likely to have similar CN preferences
	HMRF	A CN smoothing method based on hidden Markov random field with CTs as observances and CN types as hidden states
Analysis	SC	Spatial context, which is a combination of CNs such that a cell is assigned to a SC if most of its neighbors belong to a CN in the SC and such combination is minimal
	Motif	A graph with CNs as nodes connected by two kinds of edges: (1) cnX-cnY, which indicates an instance of cnX is adjacent to an instance of cnY, and (2) cnXnotY, which indicates an instance of cnX is not adjacent to any instance of cnY
	cnX → cnX-cnY	An assembly rule that indicates given cnX’s instances, most of them are adjacent to cnY’s instances
	cnX → cnXnotY	An assembly rule that indicates given cnX’s instances, most of them are not adjacent to cnY’s instances

### CN identification methods

#### CC

It represents each cell by the CT frequencies among its nearest *m* neighbors including itself and then clusters cells into CNs using *k*-means.

#### CF-IDF

It first constructs a distance-weighted *ε*-radius graph for each image with mean degree *d* and edge weight *w*_*xy*_ for each pair of cells *x* and *y* defined as

$$\begin{array}{c} w_{xy}={\text{log}}_{2}\frac{1}{0.005+\frac{dist(x,y)}{\sqrt{\sum_{\left(x,y\right)\in C^{2}}{dist(x,y)}^{2}}}}, \end{array}$$ (3)

where *dist*(⋅, ⋅) denotes the euclidean distance between cells. Communities are then detected by the Louvain algorithm [28] with resolution *r* and represented by element-wise multiplying their CT frequencies by the log inverse dataset-wise CT frequencies. Finally, it clusters communities into CNs using *k*-means.

#### CNE

It first represents each cell *x* by a vector $f\left(x\right)={\left[f_{t}\left(x\right)\right]}_{t\in T}$, where *f*_*t*_(*x*) sums up the probability densities of neighboring cells in CT *t* under a spatial Gaussian centered at *x*. The distance of *x* to itself is set as the minimum distance of *x* to other cells. The variance of the Gaussian is adapted based on the *t*-SNE’s perplexity measurement, i.e., entropy of the neighboring cells’ Gaussian densities, to alleviate the spatial cell density bias. Formally, *f*_*t*_(*x*) can be written as

$$\begin{array}{c} f_{t}\left(x\right)=\sum_{\begin{array}{c} y\in G_{x}\cup \left\{x\right\}\\ t_{y}=t \end{array}}\;\text{exp}\left(-\frac{\text{max}{\left\{dist\left(x,y\right),\underset{z\in G_{x}}{\text{min}}\;dist\left(x,z\right)\right\}}^{2}}{{\sigma_{x}}^{2}}\right)=\sum_{\begin{array}{c} y\in G_{x}\cup \left\{x\right\}\\ t_{y}=t \end{array}}q_{y|x} \end{array}$$ (4)

$$\begin{array}{c} \text{s.t.}\;p_{y|x}=\frac{q_{y|x}}{\sum_{y\in G_{x}\cup \left\{x\right\}}q_{y|x}},\;\sum_{y\in G_{x}\cup \left\{x\right\}}p_{y|x}\;\text{ln}\frac{1}{p_{y|x}}=perp, \end{array}$$ (5)

where *perp* is a hyperparameter controlling the number of effective neighbors, and here *G*_*x*_ ∪ {*x*} takes the 30 nearest neighbors, which is the largest size considered by the previous CN research [5, 8]. Next, each representation is *ℓ*_1_-normalized to get local distance-weighted CT frequencies and then element-wise multiplied by the log inverse dataset-wise CT frequencies to alleviate the overall CT distribution bias, inspired by CF-IDF. The processed *f*_*t*_(*x*) can be formally written as

$$\begin{array}{c} f_{t}\left(x\right)\leftarrow \frac{f_{t}\left(x\right)}{{\left\|f\left(x\right)\right\|}_{1}}\cdot \text{ln}\frac{1}{F(t)}, \end{array}$$ (6)

where *F*(*t*) denotes the overall frequency of CT *t* (Table 2). With these representations, CNE finally clusters cells into CNs using spatially-regularized *k*-means, which can be formulated in each iteration as

$$\begin{array}{c} c_{n}\leftarrow \frac{1}{\left|\left\{x|n_{x}=n\right\}\right|}\sum_{n_{x}=n}f\left(x\right),\;\forall n\in N, \end{array}$$ (7)

$$\begin{array}{c} n_{x}\leftarrow \underset{n\in N}{\text{arg}\;\text{min}}\;{\left\|f\left(x\right)-c_{n}\right\|}^{2}+\frac{\lambda }{\left|G_{x}\right|}\sum_{y\in G_{x}}{\left\|c_{n}-c_{n_{y}}\right\|}^{2},\;\forall x\in C. \end{array}$$ (8)

The second term in Eq 8 is added to encourage similar cluster representations among neighboring cells in order to improve CN smoothness, which works similarly as the graph Laplacian regularizer widely used in image segmentation smoothing, where λ is a hyperparameter balancing CN purity and smoothness, which was set to 0.25 in all experiments, and *G*_*x*_ considers neighbors from Delaunay triangulation.

#### Spatial LDA

It applies LDA with each cell’s CT as a “word”, each cell and its neighbors within *ε* pixels as a “document”, and each CN type as a “topic”, and introduces a prior on CN preferences of all neighborhoods $∼\text{Dirichlet}\left(\alpha_{1}\right),\text{Dirichlet}\left(\alpha_{2}\right),\cdots ,\text{Dirichlet}\left(\alpha_{\left|C\right|}\right)$ as

$$\begin{array}{c} p\left(\alpha_{1},\alpha_{2},\cdots ,\alpha_{\left|C\right|}\right)\propto \Pi_{(x,y)\in edges}\;\text{Laplace}\left(\alpha_{x}-\alpha_{y};b\right), \end{array}$$ (9)

where *edges* come from Delaunay triangulation of cell images and *b* is a hyperparameter. Briefly, the CN preference of each cell, represented by the CN preference of the neighborhood centered at it, is learned by maximizing the probability of cell’s CT occurrence in the neighborhood under the latent CN preference per neighborhood and CT preference per CN.

#### ClusterNet

It uses 2-layer graph convolutional networks to embed cells in each image (considered as a *k*-NN graph with hyperparameter *k*) and then applies differentiable soft *k*-means for clustering. It is trained by the loss—modularity, which is a measure in community detection. Global clustering is used for inference. We followed the same architecture of the original implementation, used the default hyperparameter, and stopped training when the loss no longer decreased for three epochs.

#### GAP

It uses two GraphSAGE [29] layers to embed cells in each image (considered as a *k*-NN graph with hyperparameter *k*) and then uses 2-layer MLPs followed by softmax to generate CN types. It is trained by the loss—normalized cut, which is a measure in community detection. Since no official implementation is available, we implemented it by ourselves, following the similar architecture of ClusterNet, and stopped training when the loss no longer decreased for three epochs.

### Experiment settings of CN identification

#### CRC dataset

CC (*m* = 10) was originally used to identify ten CNs in the CRC dataset with one “dirt” enriched CN removed afterwards [5]. To achieve similar effects, we instead discarded all dirt cells before CN identification, since a dirt enriched CN might not necessarily exist and CN removal could lead to losing normal cells. We then applied all CN identification methods including CC and obtained nine CNs.

#### T2D dataset

CF-IDF (*ε* = ∞, *r* = 0.5) was originally used to identify six CNs in the T2D dataset considering additional undefined cells [6]. We removed these undefined cells (43.6% of all cells) to allow methods other than CF-IDF to get proper local CT distributions. We then applied all CN identification methods including CF-IDF and obtained six CNs as well. Results of Spatial LDA with (*ε*, *b*) = (75, 0.025), (50, 0.25), (50, 2.5), (75, 2.5) were not included because of a convergence error we experienced during running its original implementation.

#### HLT dataset

CC (*m* = 20) with CT manipulation and CN post-processing was originally used to identify eleven CNs in the HLT dataset. We applied all CN identification methods including CC without post-processing and identified the same number of CNs [8]. The results of Spatial LDA were missing because it did not finish within two days. The results of ClusterNet and GAP were missing because of the out of memory error (on a 32G memory computer), caused by the huge size of the image, i.e., 421,516 cells per image on average.

### CN smoothing techniques

#### Naive Smoothing

It uses edges in Delaunay triangulation of cell images to define neighbors and utilizes cell representations to re-assign each cell in small CN instances to the CN of its neighbor that resides in a large CN instance and has the most similar representation under cosine similarity. For any cell *x*, let *f*(*x*) denote its representation. The smoothed CN type of any cell *x* can be formally written as

$$\begin{array}{c} n_{x}\leftarrow \{\begin{array}{cc} n_{x} & \text{if}\;|N_{x}|\geq s\\ n_{y}\;\text{where}\;y=\underset{y\in G_{x},\left|N_{y}\right|\geq s}{\text{arg}\;\text{min}}\frac{\left\langle f(x),f(y)\right\rangle }{\left\|f(x)\|\cdot \|f(y)\right\|} & \text{if}\;|N_{x}|<s \end{array}, \end{array}$$ (10)

where *s* was set to three in all experiments.

#### HMRF

It builds a HMRF model that takes cells as nodes, CTs as observances, and CN types as hidden states in a *ε*-radius graph with a default mean degree of five and solves it by the EM algorithm. During the expectation step, it estimates the distribution of CT given CN as

$$\begin{array}{c} P\left(CT=t|CN=n\right)=\frac{\left|\left\{x\in C|t_{x}=t,n_{x}=n\right\}\right|}{\left|\left\{x\in C|n_{x}=n\right\}\right|},\;\forall t\in T,n\in N. \end{array}$$ (11)

During the maximization step, it updates CN types by maximizing the probability of each cell’s CN type given its CT and its neighbors’ CN types, assuming each cell’s CT is only dependent on its CN type, which can be formally written as

$$\begin{array}{c} \begin{array}{cc} n_{x} & =\underset{n\in N}{\text{arg}\;\text{max}}\;P\left(CN=n|CT=t_{x},\left\{n_{y}|y\in G_{x}\right\}\right)\\ & =\underset{n\in N}{\text{arg}\;\text{max}}\;\frac{P\left(CT=t_{x}|CN=n,\left\{n_{y}|y\in G_{x}\right\}\right)\cdot P\left(CN=n|\left\{n_{y}|y\in G_{x}\right\}\right)}{P(CT=t_{x}|\left\{n_{y}|y\in G_{x}\right\})}\\ & =\underset{n\in N}{\text{arg}\;\text{max}}\;P\left(CT=t_{x}|CN=n\right)\cdot P\left(CN=n|\left\{n_{y}|y\in G_{x}\right\}\right)\\ & =\underset{n\in N}{\text{arg}\;\text{max}}\;P\left(CT=t_{x}|CN=n\right)\cdot \text{exp}(\beta \sum_{y\in G_{x}}1_{n_{y}=n}), \end{array} \end{array}$$ (12)

where *β* is a hyperparameter set to be nine as default. In this step, the new CN type of the target cell *x* is chosen by calculating the value of the probability in Eq 12 for each CN type n∈N and finding the maximizer, where the first term in Eq 12 is estimated by Eq 11 and the second term is computed by comparing each CN type n∈N with *x*’s neighboring cells’ CN types. The algorithm ends when all cells’ CN types converge.

### CN analysis methods

#### CT Enrichment

It computes a CT-CN position weight matrix (PWM) to see CT enrichments across different CNs, where the enrichment score of CT *t* in CN *n* is computed as the log ratio of the frequency of CT *t* in CN *n* to the overall frequency of CT *t*, i.e., ${\text{log}}_{2}\frac{|C_{n,t}|+F(t)}{|C_{n}|+1}-{\text{log}}_{2}\;F(t)$. CNs are then named as the most enriched CTs.

#### Differential CT Enrichment

It estimates a linear model

$$\begin{array}{c} {\text{log}}_{2}\;F_{n}^{d}\left(t\right)=\beta_{0}+\beta_{1}1_{d\in D}+\beta_{2}\;{\text{log}}_{2}\;F^{d}\left(t\right) \end{array}$$ (13)

with regard to *d* for each CT *t* and CN *n* given a target donor group D, where *F*^*d*^(*t*) denotes the frequency of CT *t* in donor *d*, $F_{n}^{d}\left(t\right)$ denotes the frequency of CT *t* in donor *d* and CN *n* (Table 2), and (*β*_0_, *β*_1_, *β*_2_) are parameters. The estimate and corresponding *p*-value of *β*_1_ can indicate the influence of the target donor group on CT enrichments in different CNs.

#### Tensor Decomposition

It builds a 3D tensor $X\in R^{\left|T|\times |N|\times |D\right|}$ for the target donor group D by stacking donor-specific CT-CN joint distribution matrices in D and then decomposes it using non-negative Tucker tensor decomposition [30]. The process can be written as

$$\begin{array}{c} X\approx G\times_{1}\text{A}\times_{2}\text{B}\times_{3}\text{C}=\Sigma_{p=1}^{P}\Sigma_{q=1}^{Q}\Sigma_{r=1}^{R}g_{pqr}{\text{a}}_{p}⊗{\text{b}}_{q}⊗{\text{c}}_{r}, \end{array}$$ (14)

where $G\in R^{P\times Q\times R}$ with (*P*, *Q*, *R*) as hyperparameters is the core tensor, ⊗ denotes outer product, ${\text{a}}_{p}\in R^{\left|T\right|}$ is a factor in CT space (i.e., a CT module), ${\text{b}}_{q}\in R^{\left|N\right|}$ is a factor in CN space (i.e., a CN module), and *g*_*pqr*_ measures the interaction strength between **a**_*p*_ and **b**_*q*_ in the *r*-th slice of G (i.e., the *r*-th tissue module). In our experiments, (*P*, *Q*, *R*) was set to be (6, 6, 2) following the original study.

#### Inter-CN Communication Network

It applies canonical correlation analysis [31, CCA] to each pair of CNs (*n*_1_, *n*_2_) in the target donor group D using donor-and-CN-specific frequencies of target CTs $T_{0}$, i.e., $\text{X}={\left(F_{n_{1}}^{d}\left(t\right)\right)}_{d\in D,t\in T_{0}}\in R^{\left|D\right|\times \left|T_{0}\right|}$ and $\text{Y}={\left(F_{n_{2}}^{d}\left(t\right)\right)}_{d\in D,t\in T_{0}}\in R^{\left|D\right|\times \left|T_{0}\right|}$, and then connects (*n*_1_, *n*_2_) in the network if their largest canonical correlation is significant under the permutation test (*p* < 0.1), with the [0, 1]-normalized 1 − *p* value as the communication strength between CNs.

#### CN Combination Map

It identifies spatial contexts (SCs), sets of CNs, such that a cell is assigned to a SC if more than *x* = 90% of its nearest *y* = 100 cells are assigned to one of those CNs and if it is the minimal set of CNs that has this property, and then generates a tree-structured SC map where each SC contains at least 0.001% of total cells and is labeled by the number of cells in it.

#### Assembly Rule Identification

It constructs a graph for each image, whose nodes represent CNs and edges indicate adjacency of CN instances detected by finding connected components in Boolean images, and then identifies assembly rules that can extend at least *x* = 70% of the instances of the source motif with at least five instances to the target motif.

#### Visualization of CNs

We visualized CNs produced by different methods by projecting them onto original data in the form of Voronoi diagrams (Figs A, B, H, I, O, and R in S1 Text). We have added the visualization function as visualize.ipynb in our codebase. For more advanced visualization, we recommend users explore tools such as SPIAT [32], Scimap [33], and Vitessce [34]. The latter has been deployed for multiple spatial data types in the HuBMAP Data Portal. Other file formats such as OME-NGFF [35] can be viewed in visualization clients such as Napari [36].

## Supporting information

S1 Data Excel spreadsheet containing, in separate sheets, the underlying numerical values for generating Fig 2A, 2B, 2C, 3A, 3B, 3C, 3D, 4A, 4B, 4C, 4D, 5A, and 5B. (XLSX)

S1 Text Supporting tables A–C and figures A–V. (PDF)
