# Supplementary material for: CNTools: A computational toolbox for cellular neighborhood analysis from multiplexed images
Source: PLoS Comput Biol. 2024 Aug 28;20(8):e1012344. doi: 10.1371/journal.pcbi.1012344 (PMC11355562; doi:10.1371/journal.pcbi.1012344)
Supplement: S1 Text — (PDF) [file pcbi.1012344.s002.pdf]

Supporting information

Supporting tables

Table A: **Experimented hyperparameters and smoothing techniques for each CN identification method on the CRC dataset.** The selected ones are highlighted.

Method	Hyperparameter	Smoothing
CC	$m \in \{5, 8, \mathbf{10}, 15, 20\}$	None, **Naive**, HMRF
CF-IDF	$\varepsilon \in \{\mathbf{32}, 40, 50\}(d \in \{\mathbf{3}, 5, 8\})$, $r \in \{0.5, \mathbf{0.8}, 1.0\}$	None, **Naive**, HMRF
CNE	$perp \in \{5, 10, \mathbf{15}, 20, 25\}, \lambda = 0.25$	None, **Naive**, HMRF
Spatial LDA	$\varepsilon \in \{\mathbf{50}, 75, 100\}$, $b \in \{\mathbf{0.025}, 0.25, 2.5\}$	None, **Naive**, HMRF
ClusterNet	$k \in \{3, \mathbf{6}, 9, 12, 15\}$	None, **Naive**, HMRF
GAP	$k \in \{3, 6, 9, 12, 15\}$	None, Naive, HMRF

Table B: **Experimented hyperparameters and smoothing techniques of CN identification methods on the T2D dataset.** The selected ones are highlighted.

Method	Hyperparameter	Smoothing
CC	$m \in \{\mathbf{5}, 8, 10, 15, 20\}$	None, Naive, **HMRF**
CF-IDF	$\varepsilon \in \{35, 45, \mathbf{57}\}(d \in \{3, 5, \mathbf{8}\})$, $r \in \{\mathbf{0.5}, 0.8, 1.0\}$	None, **Naive**, HMRF
CNE	$perp \in \{5, 10, \mathbf{15}, 20, 25\}, \lambda = 0.25$	None, **Naive**, HMRF
Spatial LDA	$\varepsilon \in \{50, 75, \mathbf{100}\}$, $b \in \{0.025, \mathbf{0.25}, 2.5\}$	None, **Naive**, HMRF
ClusterNet	$k \in \{3, \mathbf{6}, 9, 12, 15\}$	None, Naive, **HMRF**
GAP	$k \in \{3, 6, 9, 12, 15\}$	None, Naive, HMRF

Table C: **Experimented hyperparameters and smoothing techniques of CN identification methods on the HLT dataset.** The selected ones are highlighted.

Method	Hyperparameter	Smoothing
CC	$m \in \{10, 15, \mathbf{20}, 25, 30\}$	None, **Naive**, HMRF
CF-IDF	$\varepsilon \in \{\mathbf{23}, 29, 36\}(d \in \{\mathbf{3}, 5, 8\})$, $r \in \{0.5, \mathbf{0.8}, 1.0\}$	None, **Naive**, HMRF
CNE	$perp \in \{5, 10, \mathbf{15}, 20, 25\}, \lambda = 0.25$	None, **Naive**, HMRF
Spatial LDA	$\varepsilon \in \{50, 75, 100\}$, $b \in \{0.025, 0.25, 2.5\}$	-
ClusterNet	$k \in \{3, 6, 9, 12, 15\}$	-
GAP	$k \in \{3, 6, 9, 12, 15\}$	-

Supporting figures for the CRC dataset

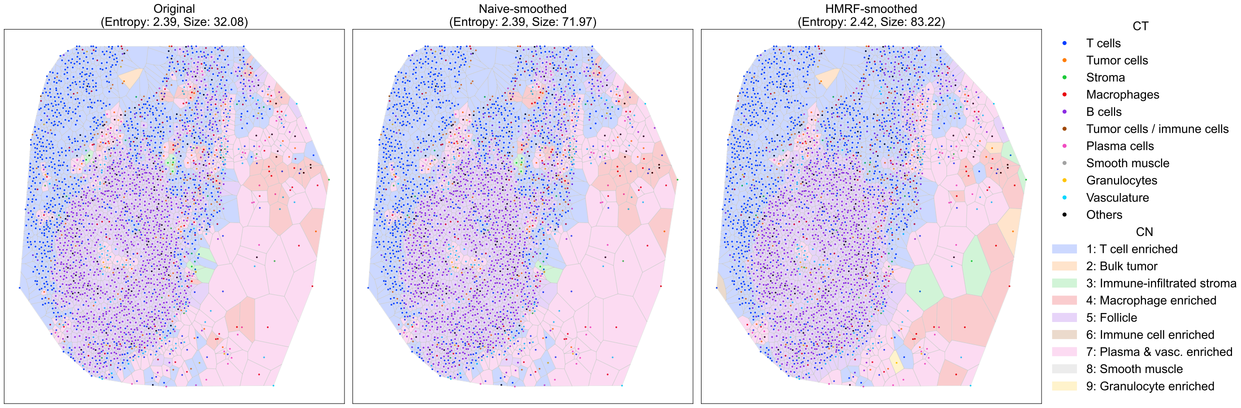

(a) CC

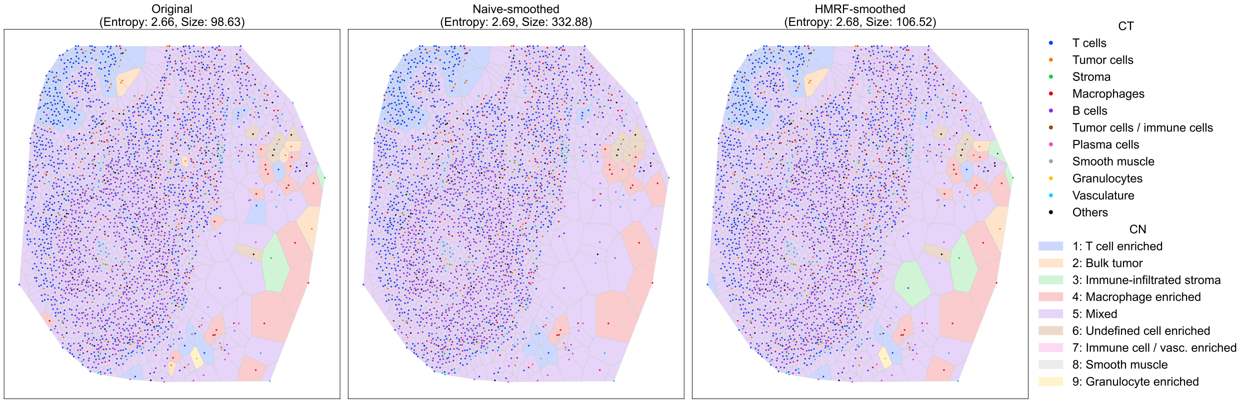

(b) CF-IDF

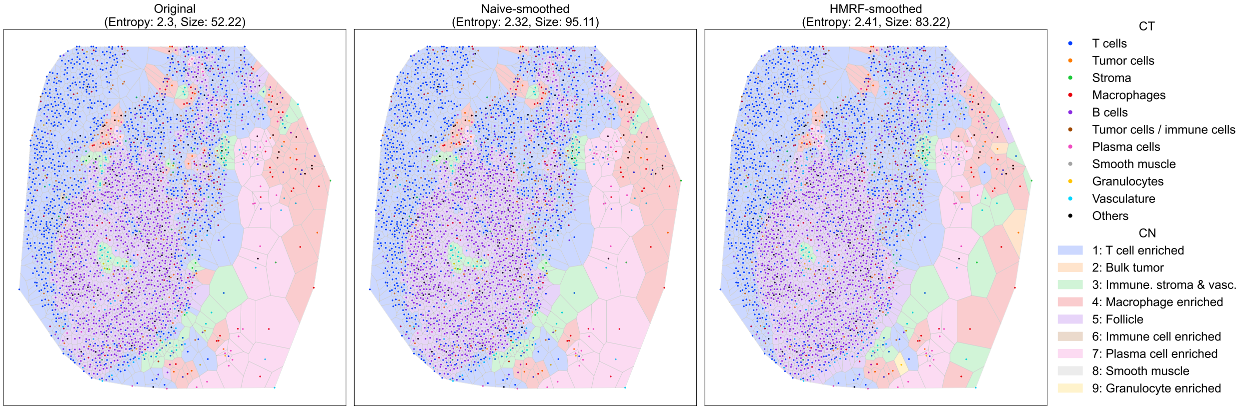

(c) CNE

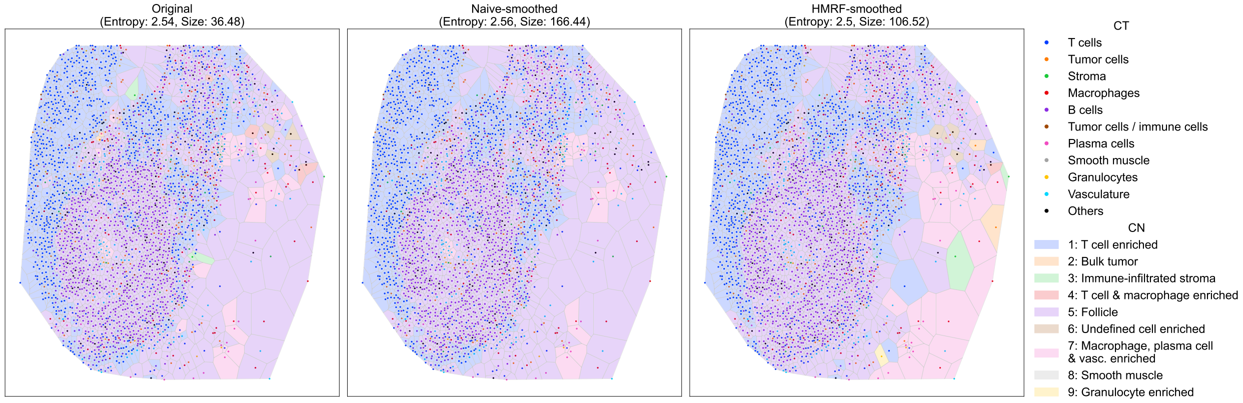

(d) Spatial LDA

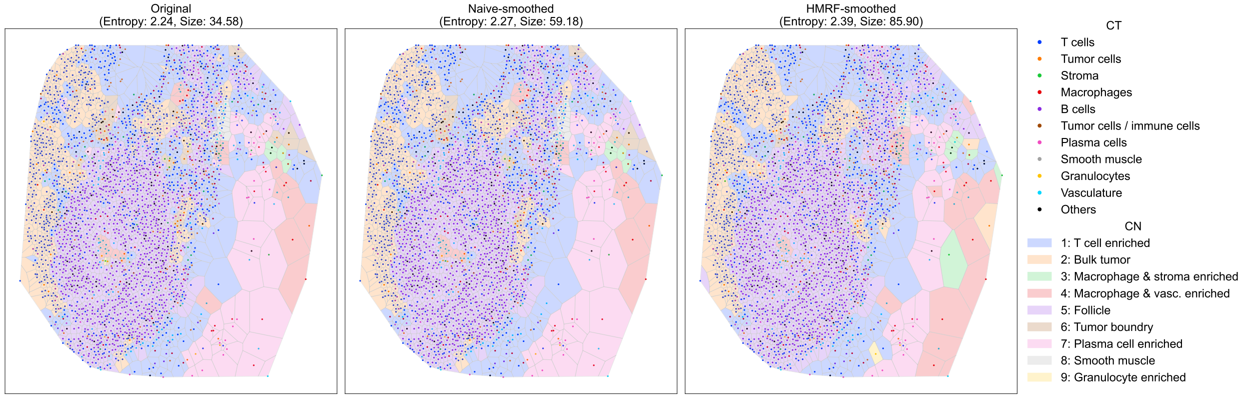

(e) ClusterNet

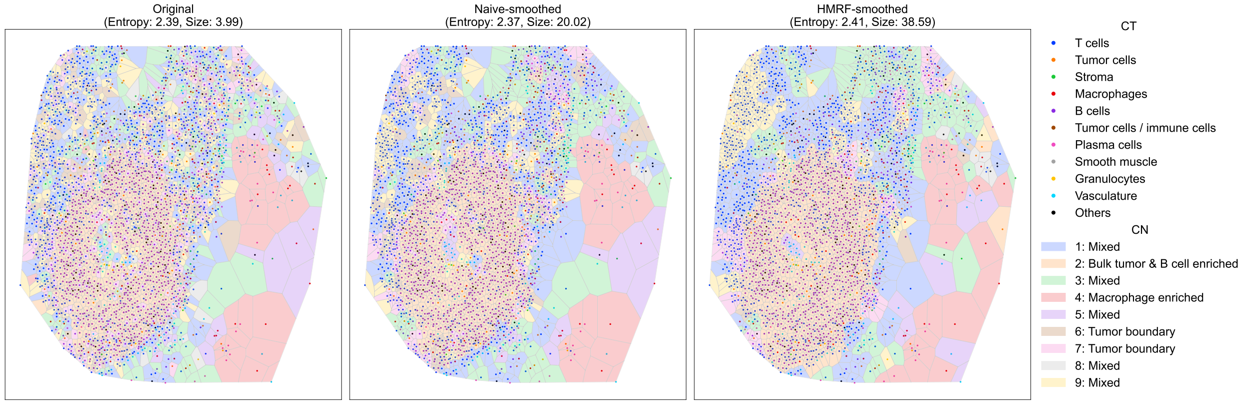

(f) GAP

Figure A: **Voronoi diagrams showing the identified CNs of different methods on image reg021_A of the CRC dataset.** For GAP, we used k=6 and Naive smoothing for illustration.

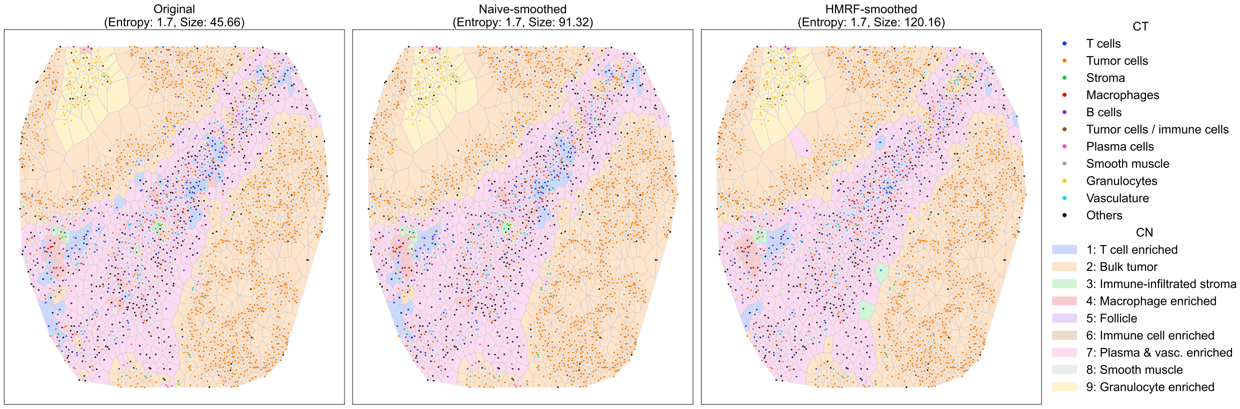

(a) CC

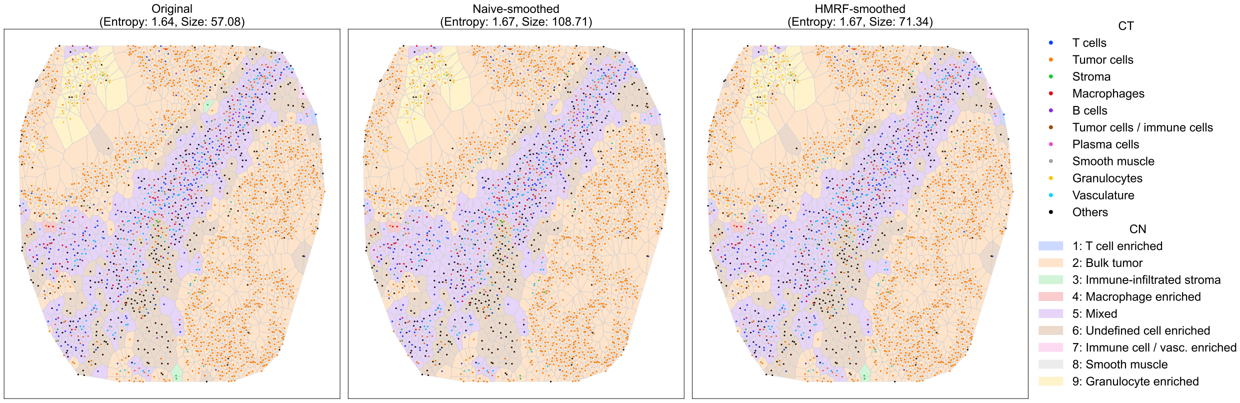

(b) CF-IDF

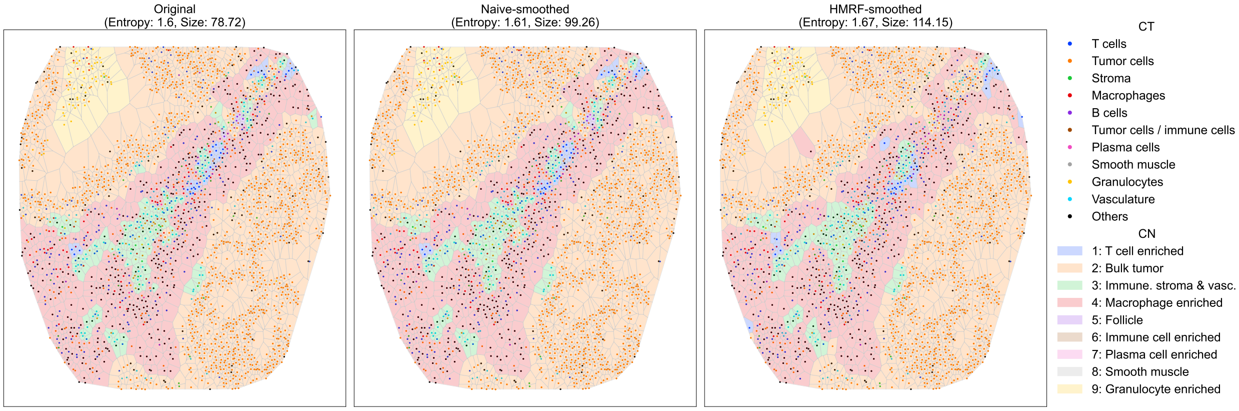

(c) CNE

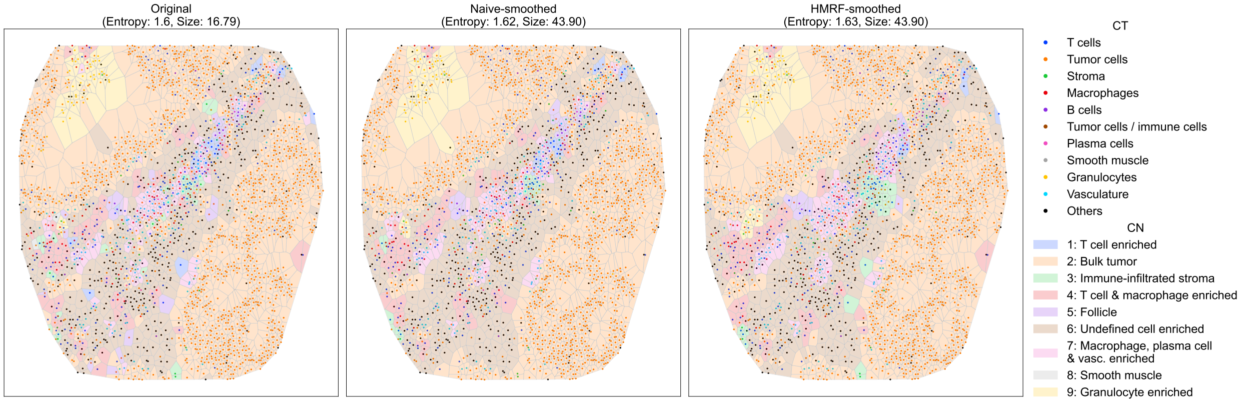

(d) Spatial LDA

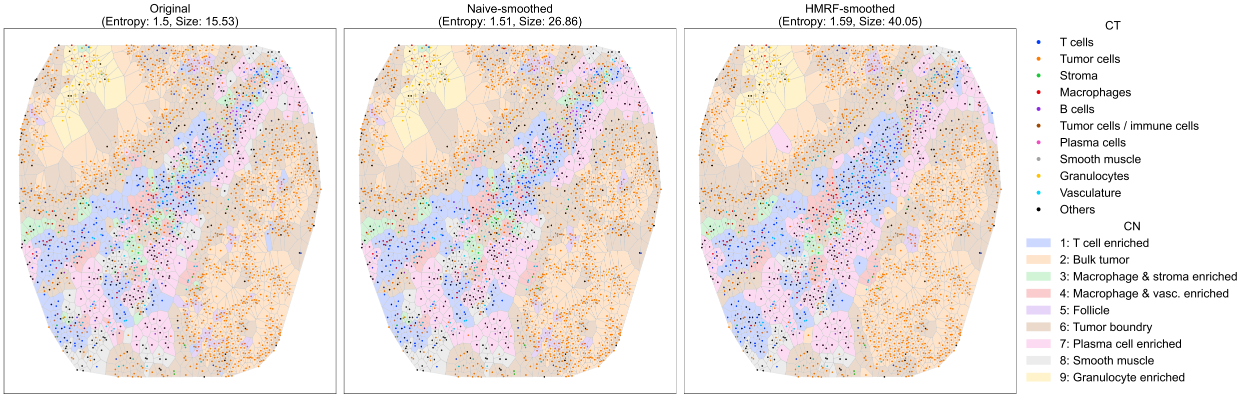

(e) ClusterNet

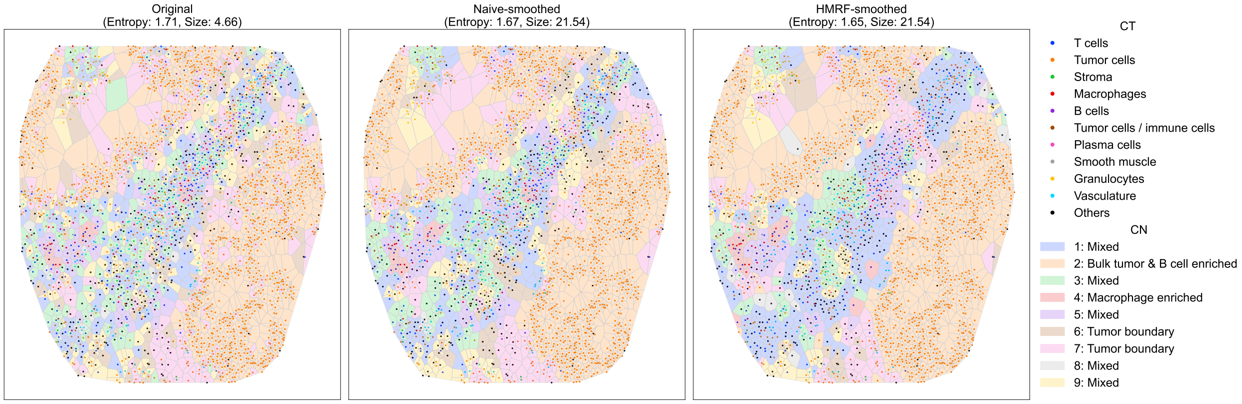

(f) GAP

Figure B: **Voronoi diagrams showing the identified CNs of different methods on image reg065_A of the CRC dataset.** For GAP, we used k=6 and Naive smoothing for illustration.

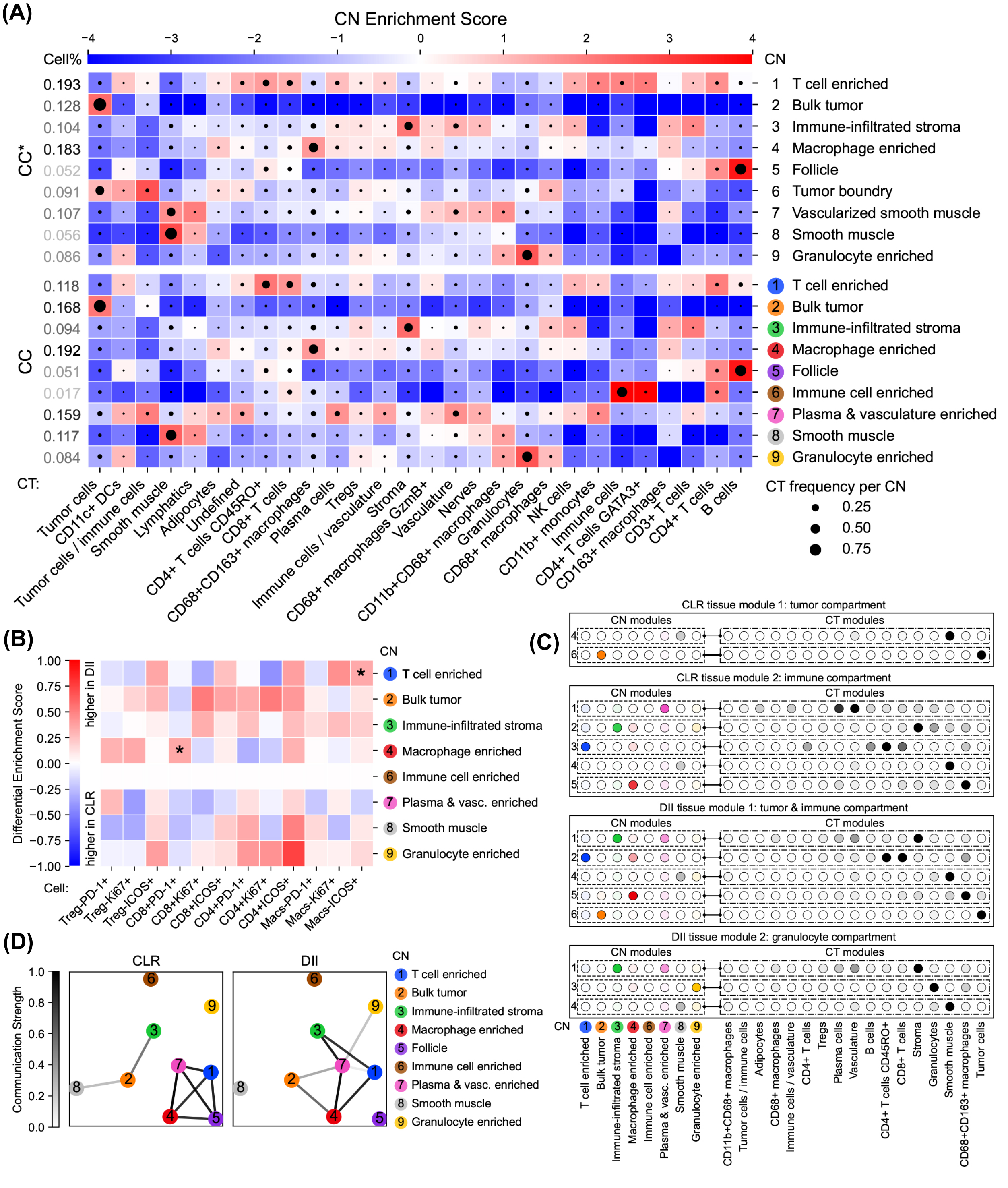

Figure C: **CN analysis results of CC on the CRC dataset.** (A) CT Enrichment analysis. (B) Differential CT Enrichment analysis. (C) Tensor Decomposition analysis. (D) Inter-CN Communication Network analysis involving {PD1+, Ki-67+, ICOS+}CD8+ T cells and Ki-67+ Tregs.

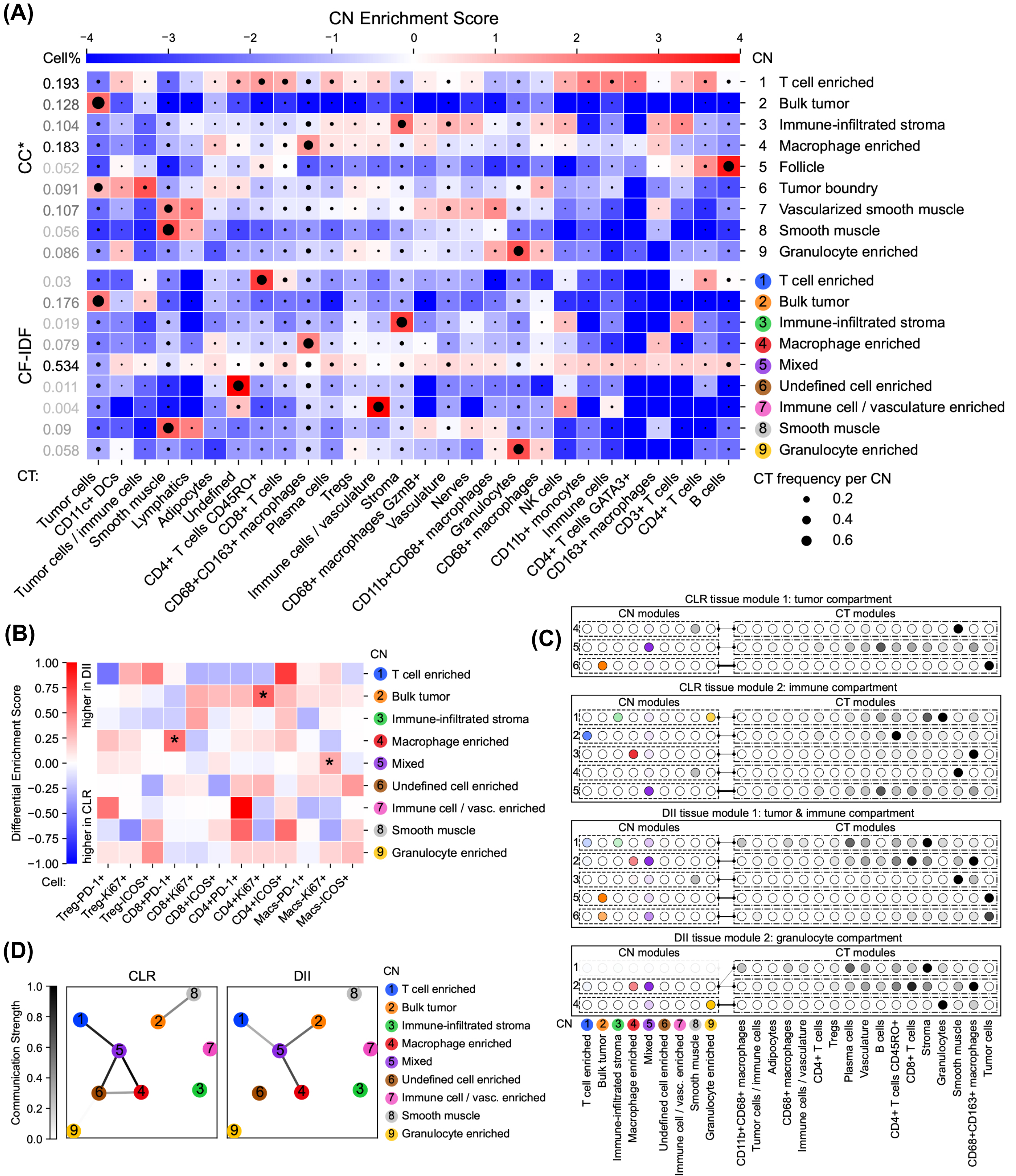

Figure D: **CN analysis results of CF-IDF on the CRC dataset.** (A) CT Enrichment analysis. (B) Differential CT Enrichment analysis. (C) Tensor Decomposition analysis. (D) Inter-CN Communication Network analysis involving {PD1+, Ki-67+, ICOS+}CD8+ T cells and Ki-67+ Tregs.

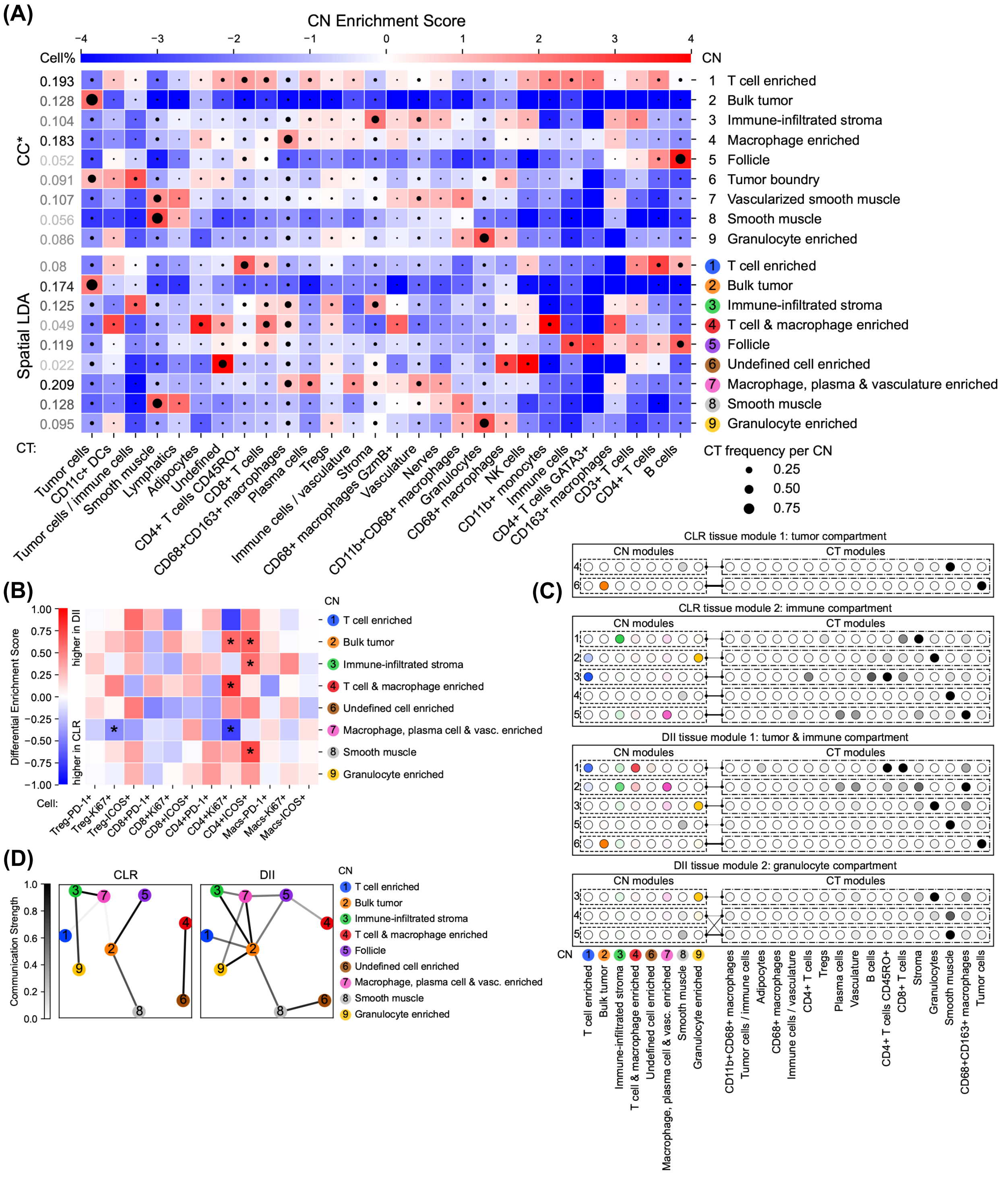

Figure E: **CN analysis results of Spatial LDA on the CRC dataset.** (A) CT Enrichment analysis. (B) Differential CT Enrichment analysis. (C) Tensor Decomposition analysis. (D) Inter-CN Communication Network analysis involving {PD1+, Ki-67+, ICOS+}CD8+ T cells and Ki-67+ Tregs.

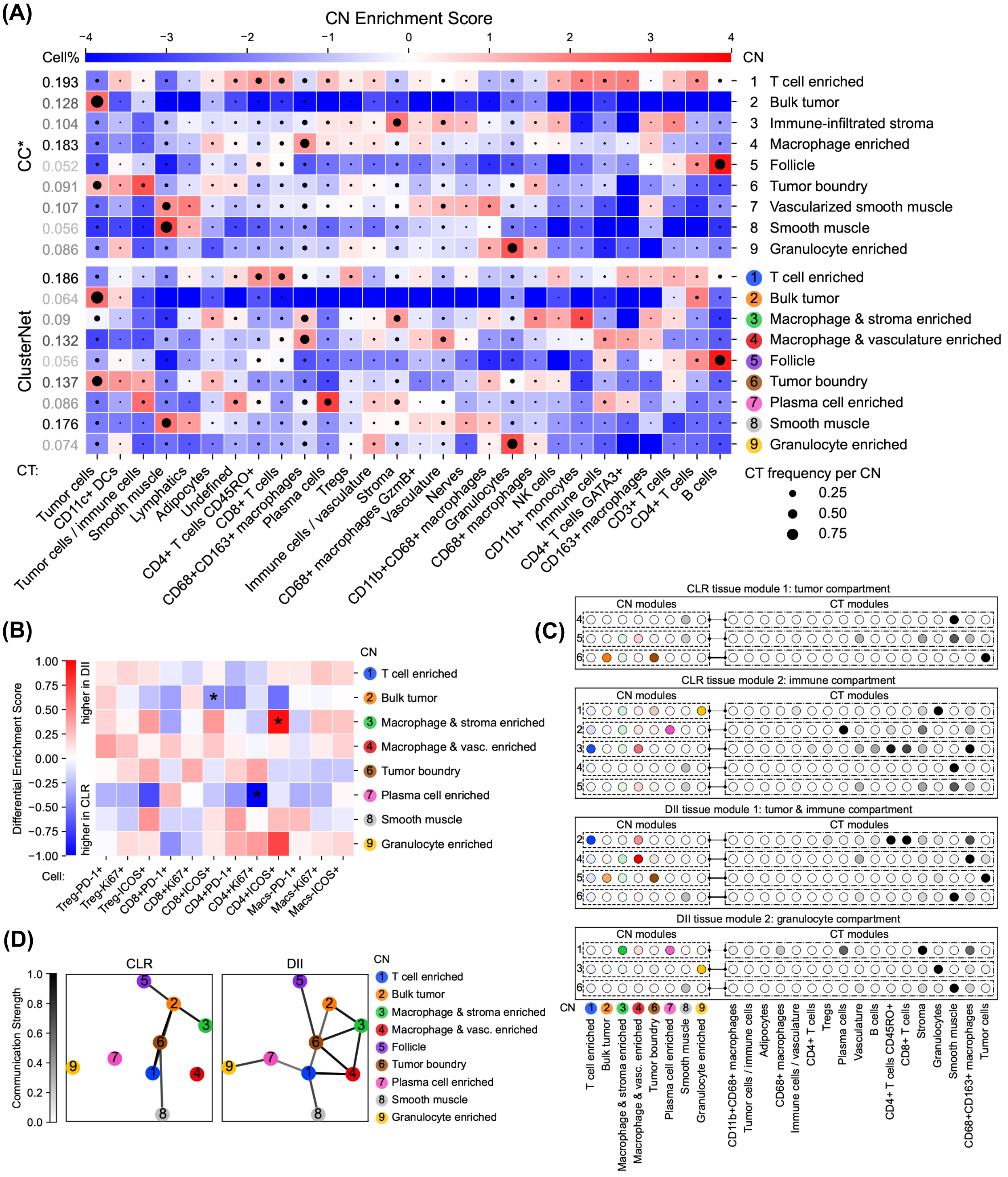

Figure F: **CN analysis results of ClusterNet on the CRC dataset.** (A) CT Enrichment analysis. (B) Differential CT Enrichment analysis. (C) Tensor Decomposition analysis. (D) Inter-CN Communication Network analysis involving {PD1+, Ki-67+, ICOS+}CD8+ T cells and Ki-67+ Tregs.

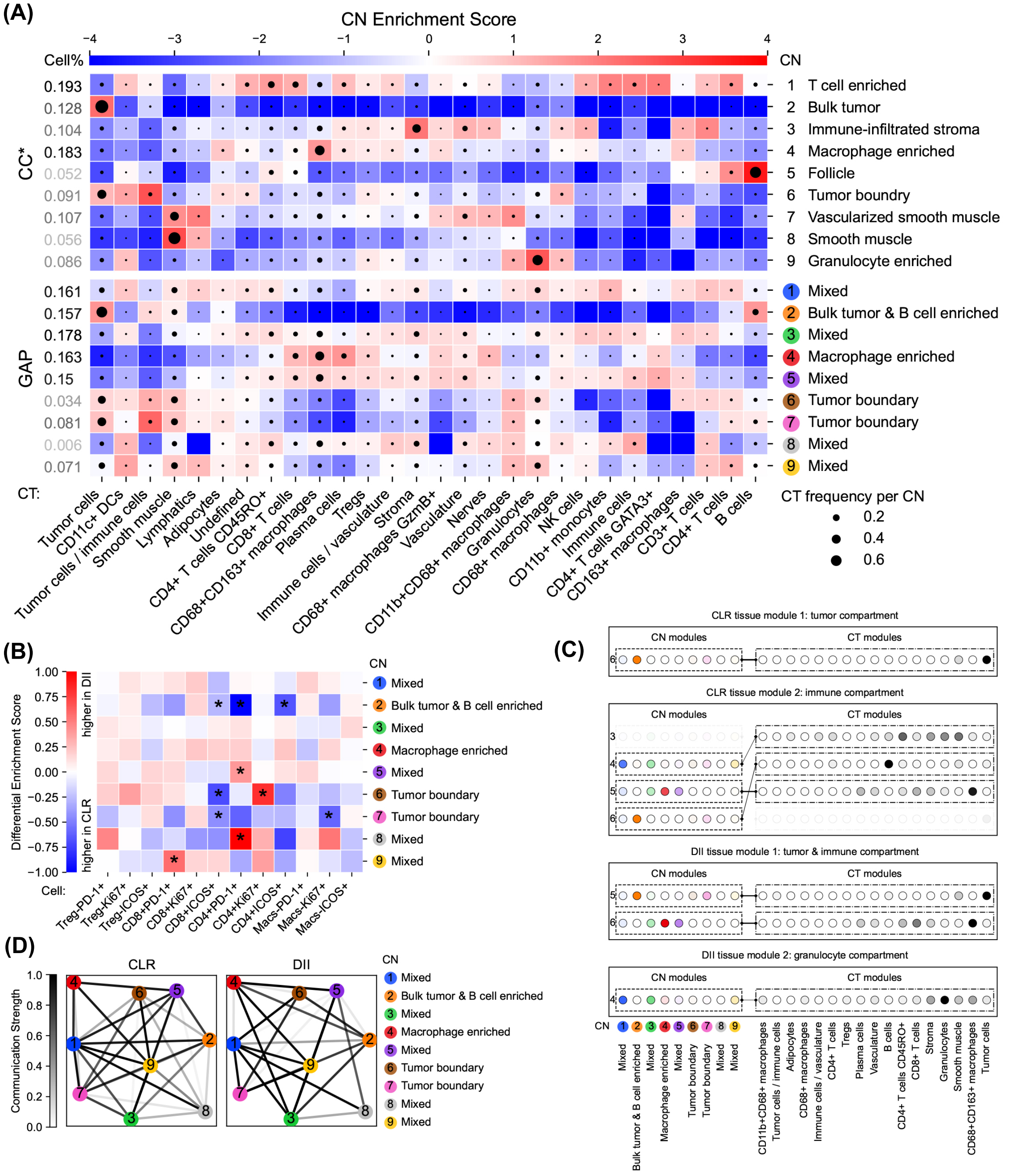

Figure G: **CN analysis results of GAP on the CRC dataset.** (A) CT Enrichment analysis. (B) Differential CT Enrichment analysis. (C) Tensor Decomposition analysis. (D) Inter-CN Communication Network analysis involving {PD1+, Ki-67+, ICOS+}CD8+ T cells and Ki-67+ Tregs.

Supporting figures for the T2D dataset

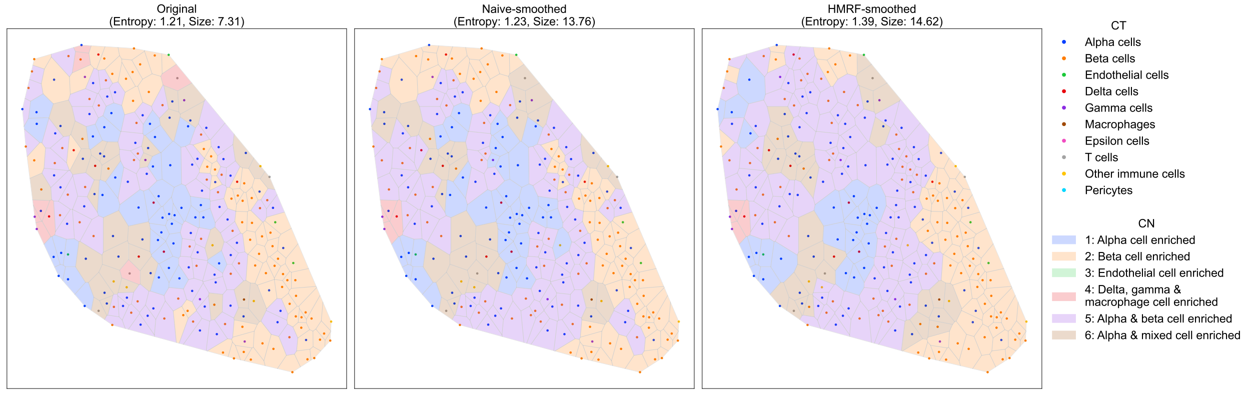

(a) CC

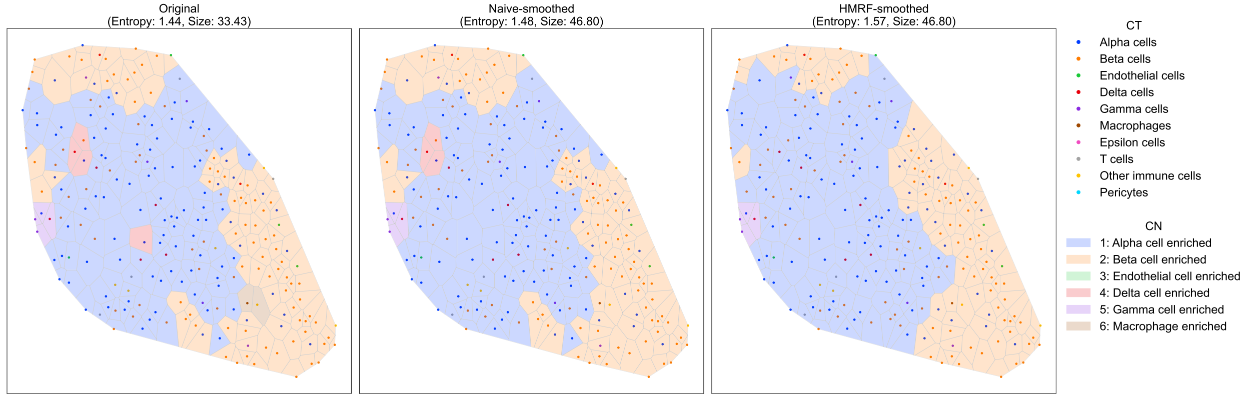

(b) CNE

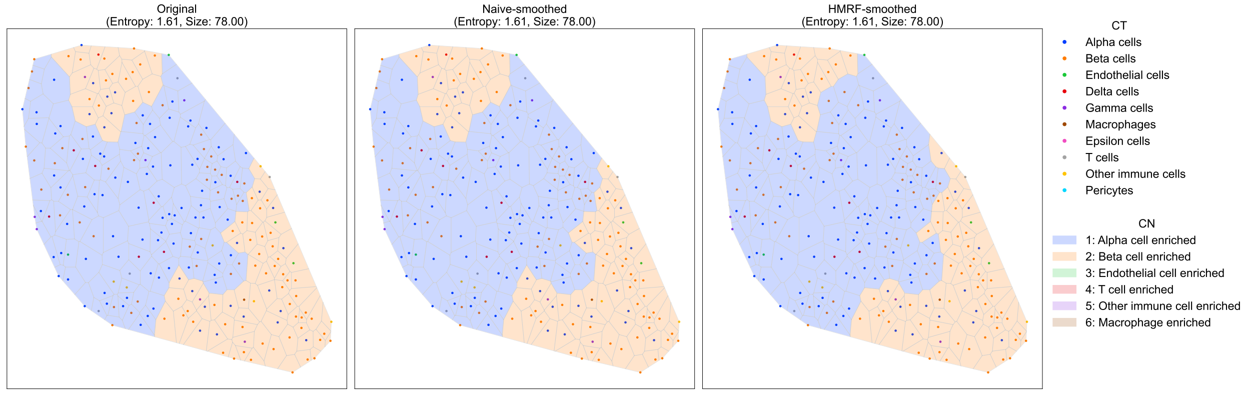

(c) CF-IDF

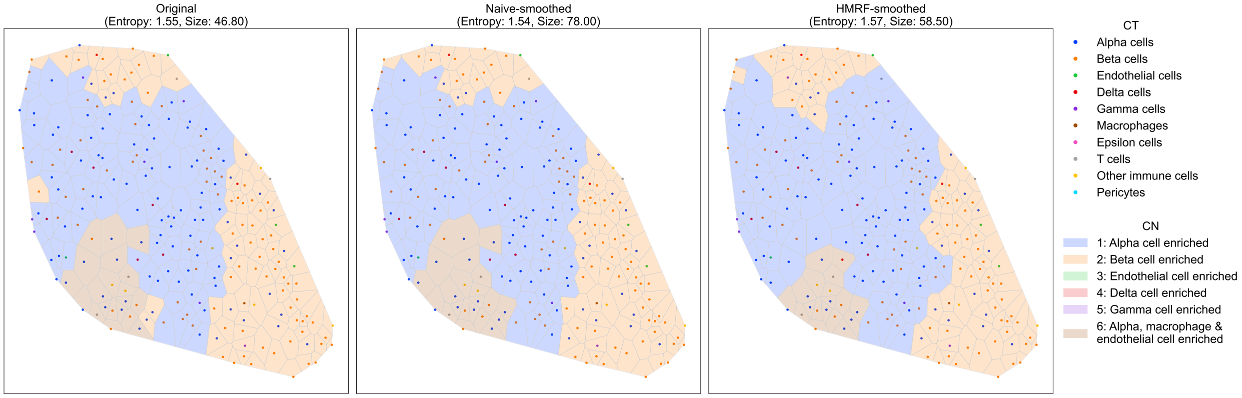

(d) Spatial LDA

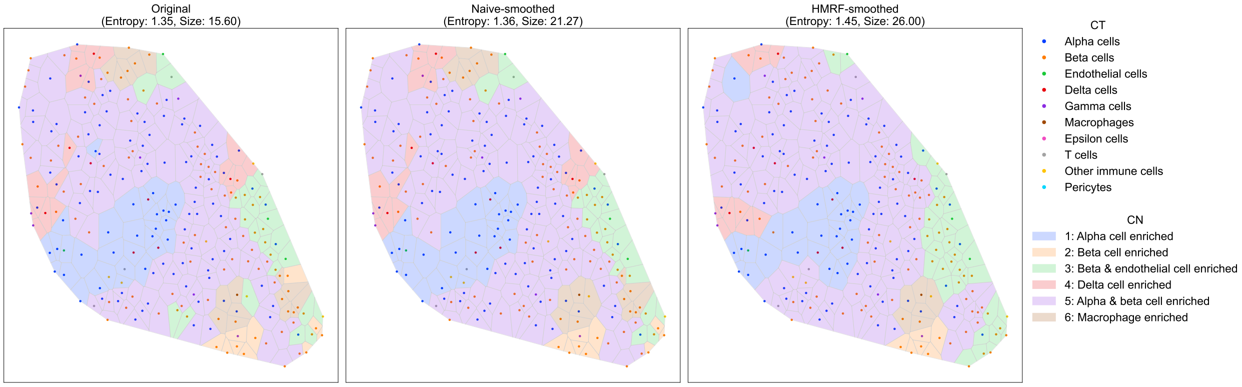

(e) ClusterNet

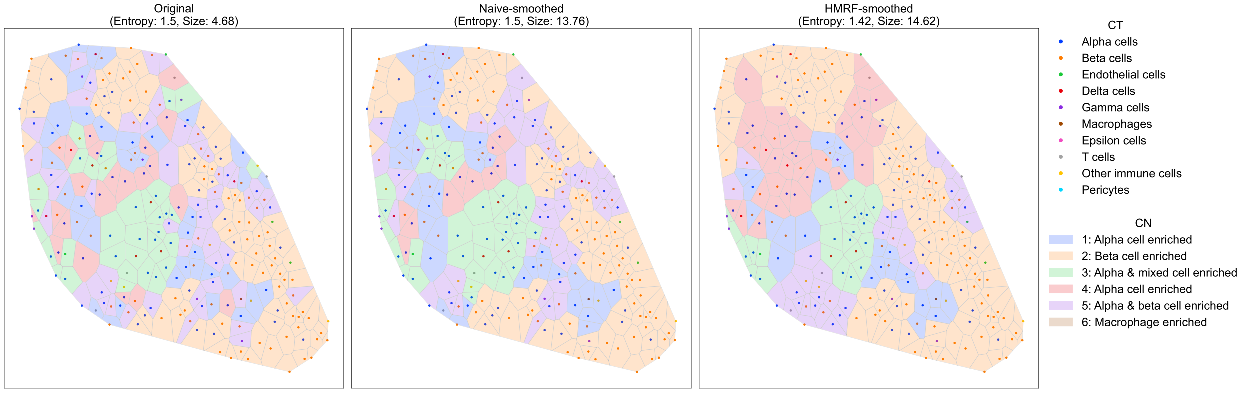

(f) GAP

Figure H: **Voronoi diagrams showing the identified CNs of different methods on image ABHQ115_2 of the T2D dataset.** For GAP, we used k=6 and HMRF smoothing for illustration.

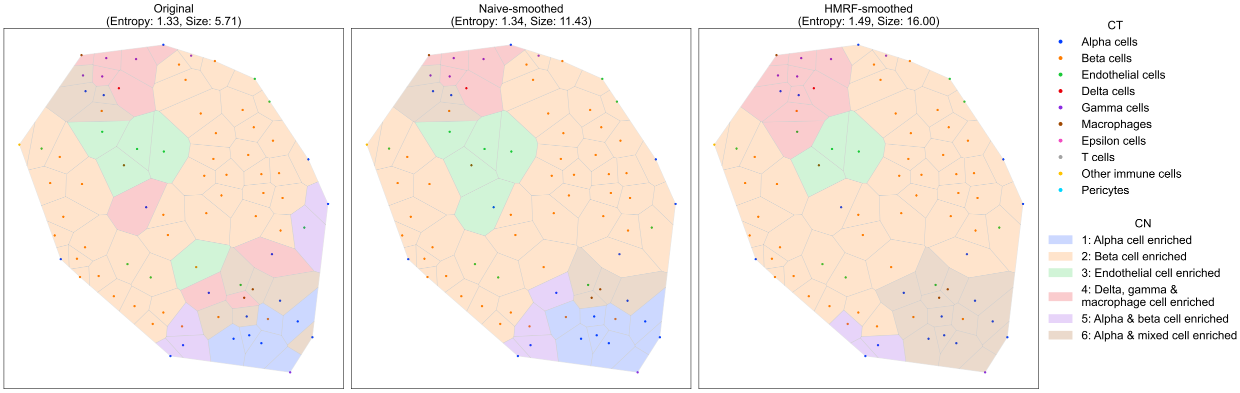

(a) CC

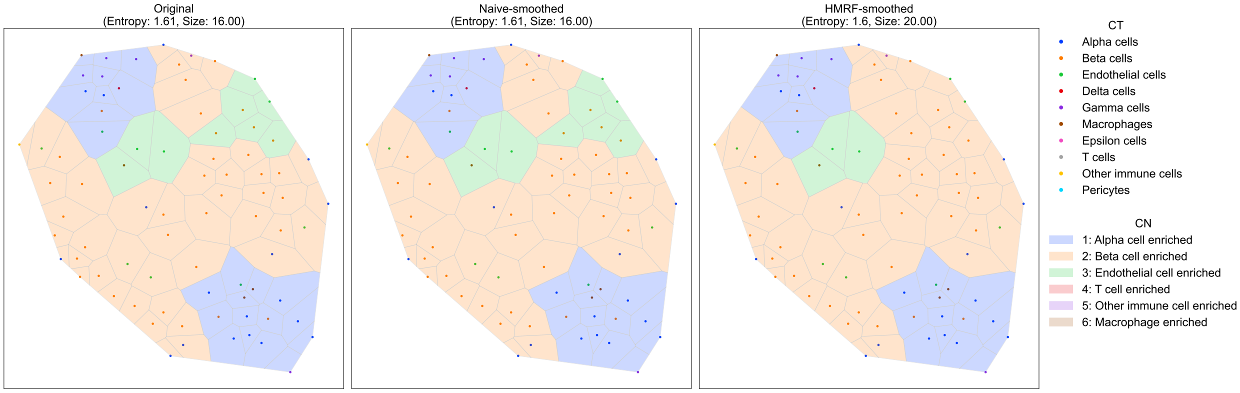

(b) CF-IDF

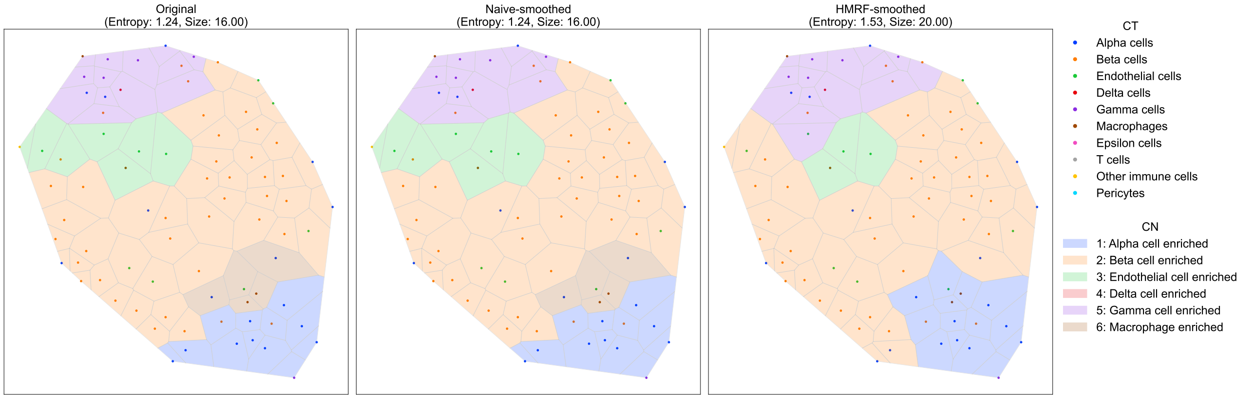

(c) CNE

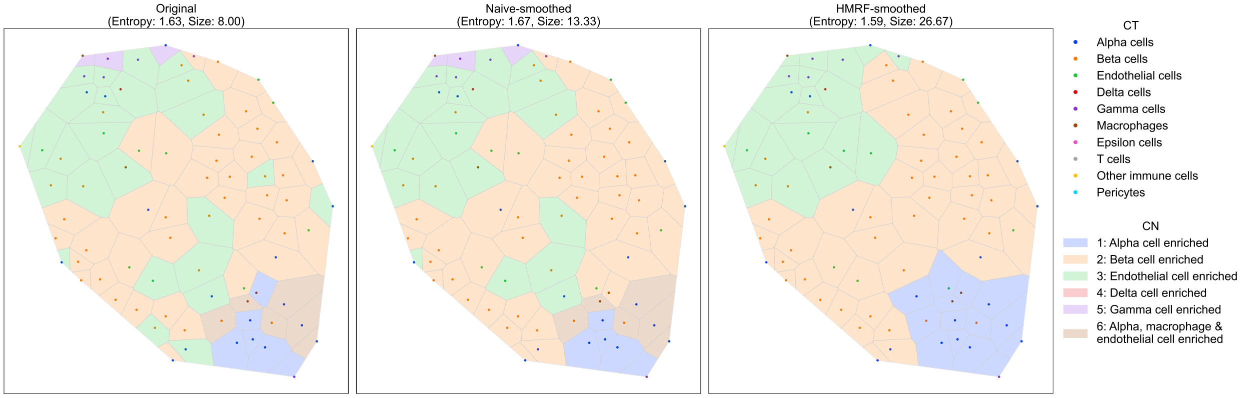

(d) Spatial LDA

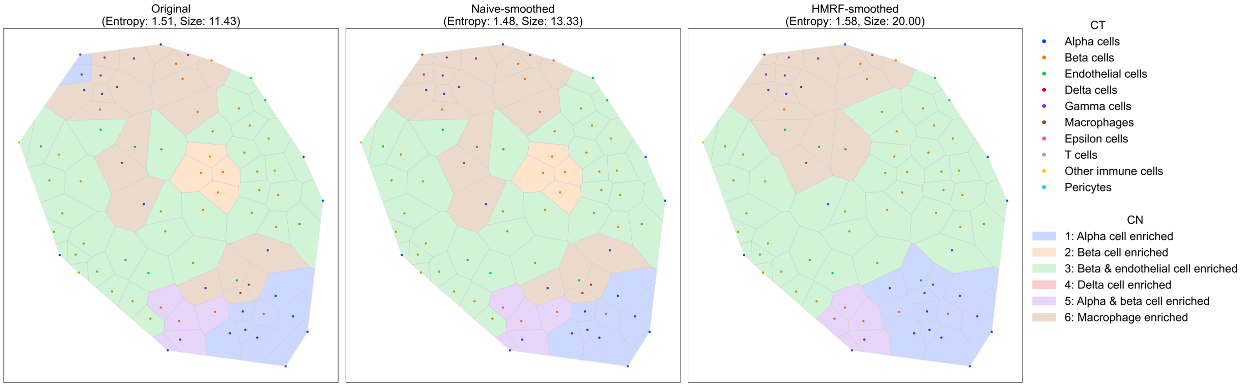

(e) ClusterNet

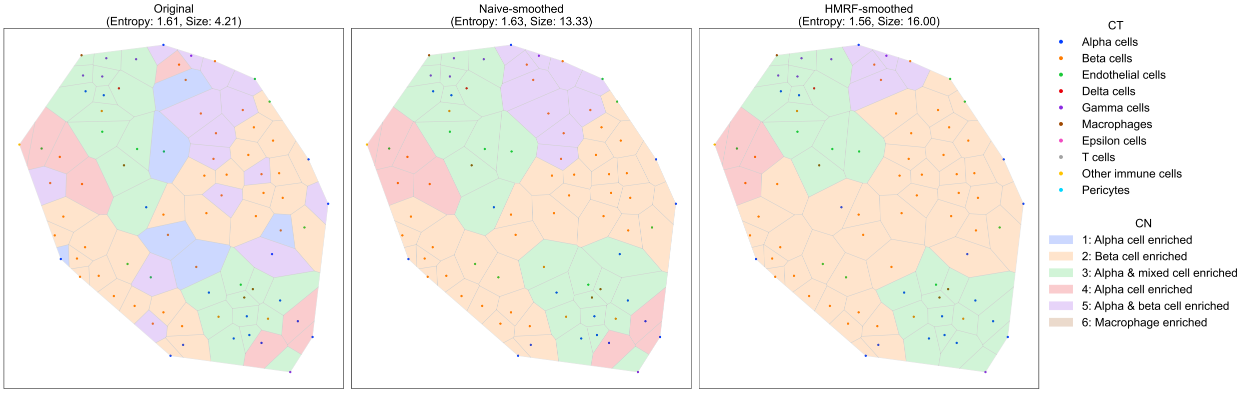

(f) GAP

Figure I: **Voronoi diagrams showing the identified CNs of different methods on image AGBA390_4 of the T2D dataset.** For GAP, we used k=6 and HMRF smoothing for illustration..

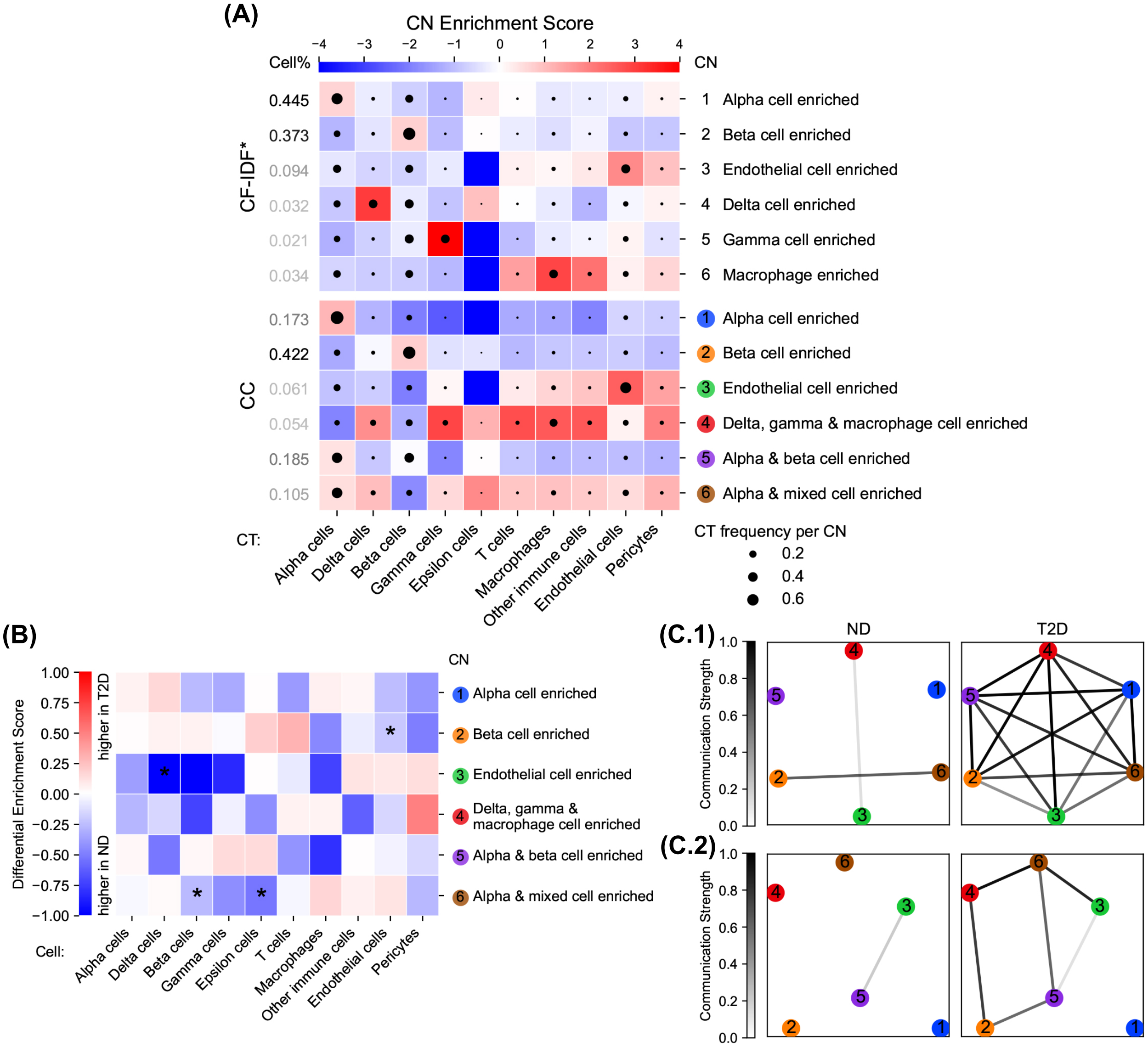

Figure J: **CN analysis results of CC on the T2D dataset.** (A) CT Enrichment analysis. (B) Differential CT Enrichment analysis. (C.1) Inter-CN Communication Network analysis involving vascular cells (endothelial cells and pericytes). (C.2) Inter-CN Communication Network analysis involving immune cells (T cells, macrophages, and other immune cells).

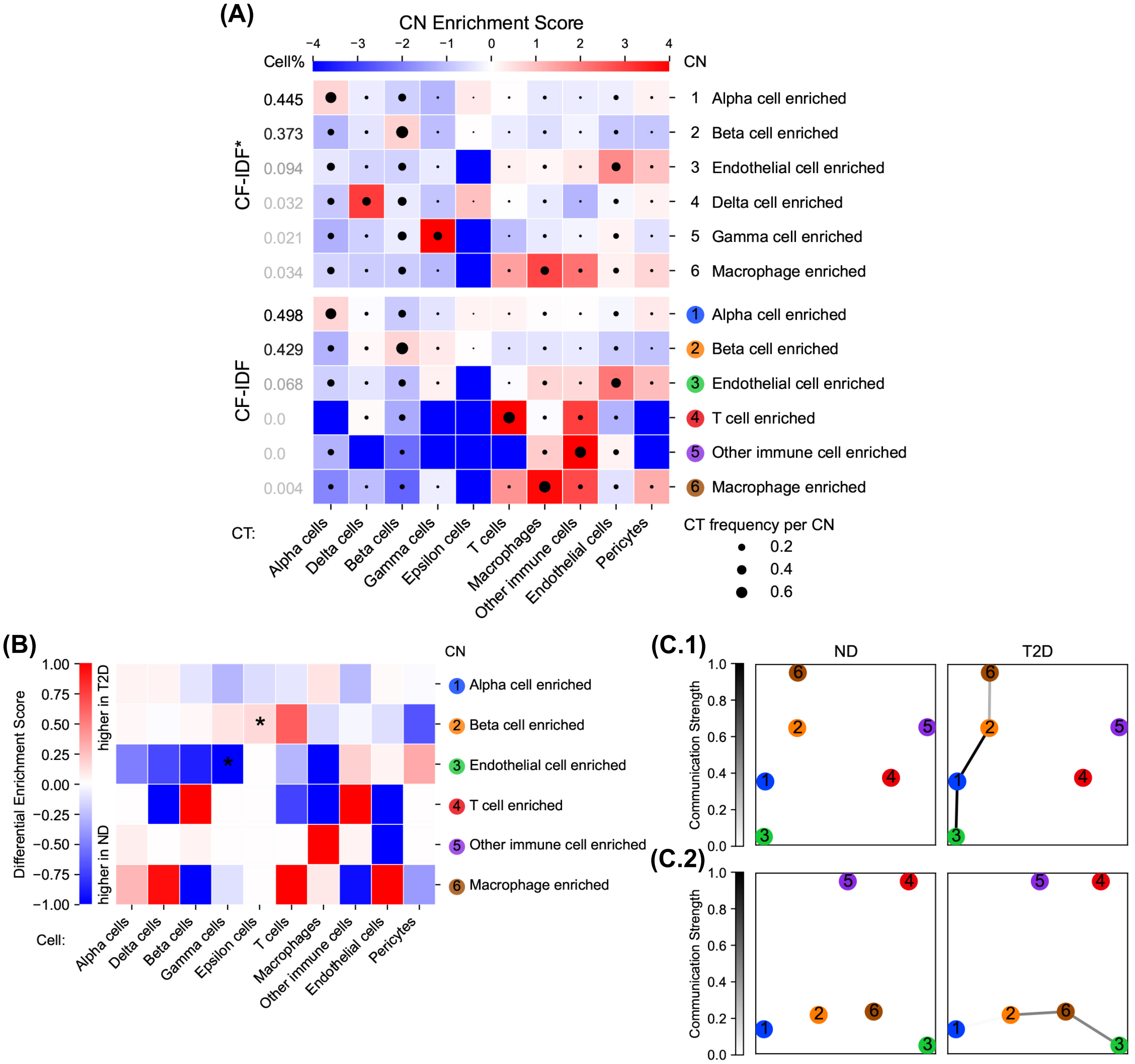

Figure K: **CN analysis results of CF-IDF on the T2D dataset.** (A) CT Enrichment analysis. (B) Differential CT Enrichment analysis. (C.1) Inter-CN Communication Network analysis involving vascular cells (endothelial cells and pericytes). (C.2) Inter-CN Communication Network analysis involving immune cells (T cells, macrophages, and other immune cells).

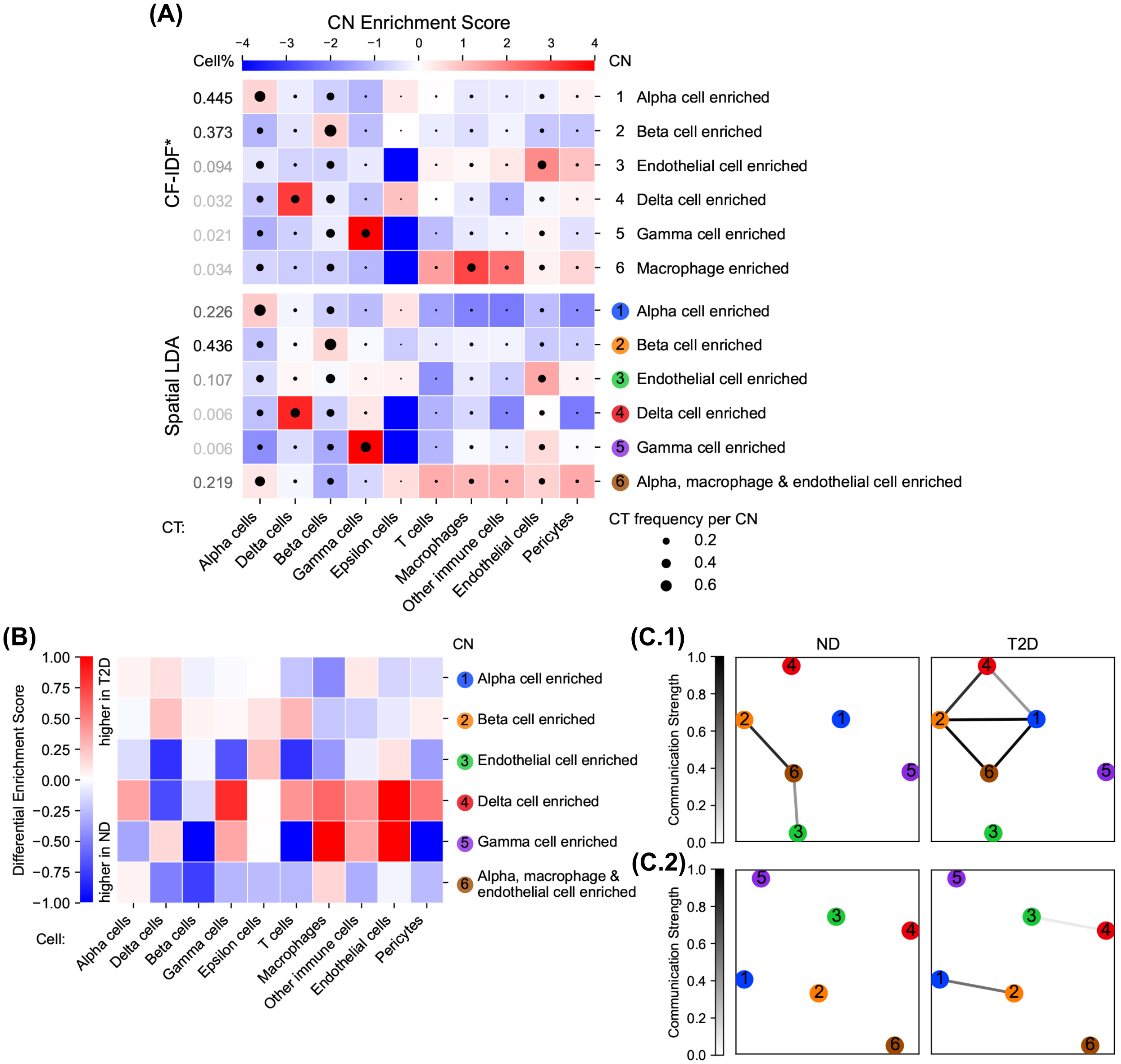

Figure L: **CN analysis results of Spatial LDA on the T2D dataset.** (A) CT Enrichment analysis. (B) Differential CT Enrichment analysis. (C.1) Inter-CN Communication Network analysis involving vascular cells (endothelial cells and pericytes). (C.2) Inter-CN Communication Network analysis involving immune cells (T cells, macrophages, and other immune cells).

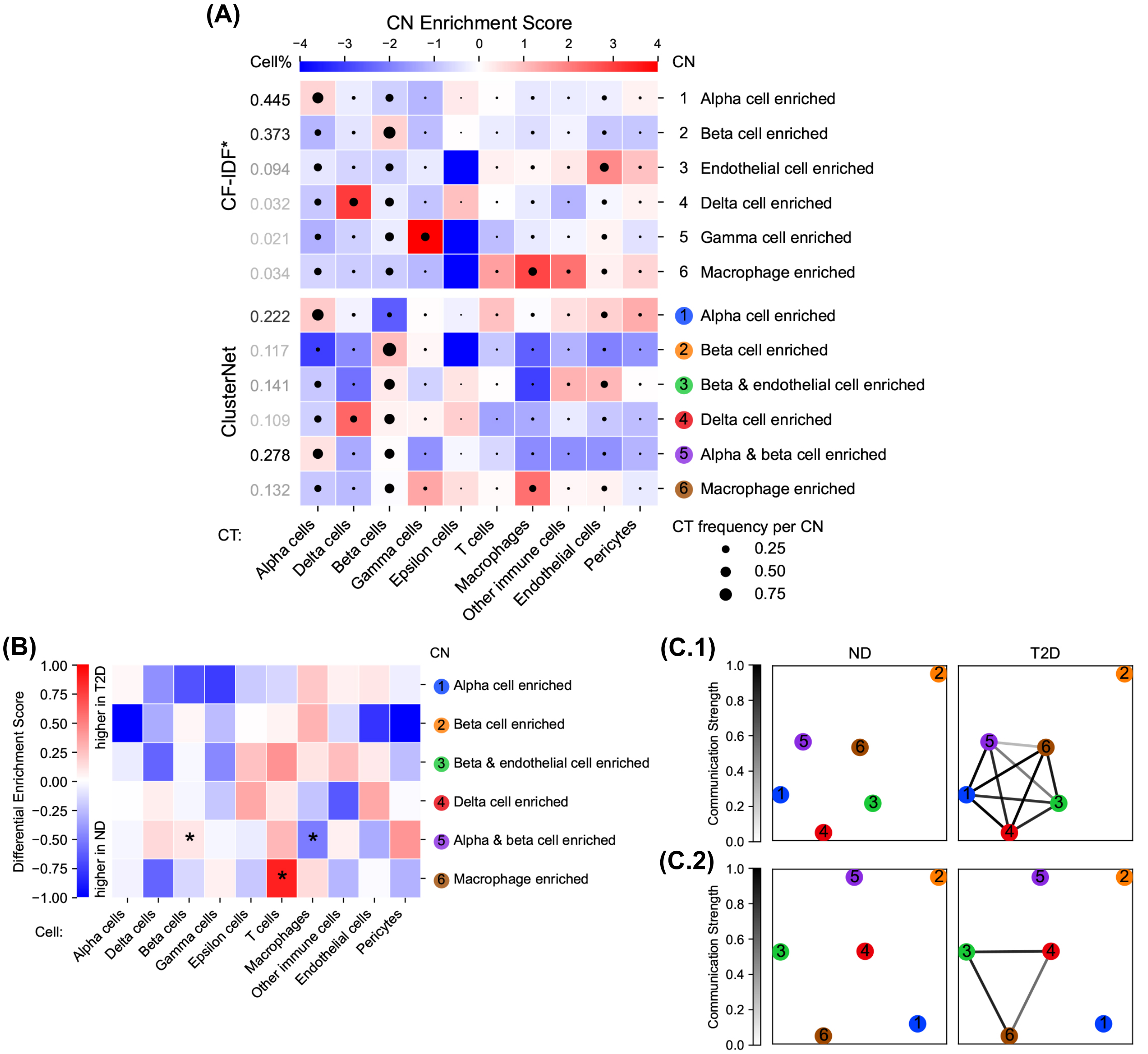

Figure M: **CN analysis results of ClusterNet on the T2D dataset.** (A) CT Enrichment analysis. (B) Differential CT Enrichment analysis. (C.1) Inter-CN Communication Network analysis involving vascular cells (endothelial cells and pericytes). (C.2) Inter-CN Communication Network analysis involving immune cells (T cells, macrophages, and other immune cells).

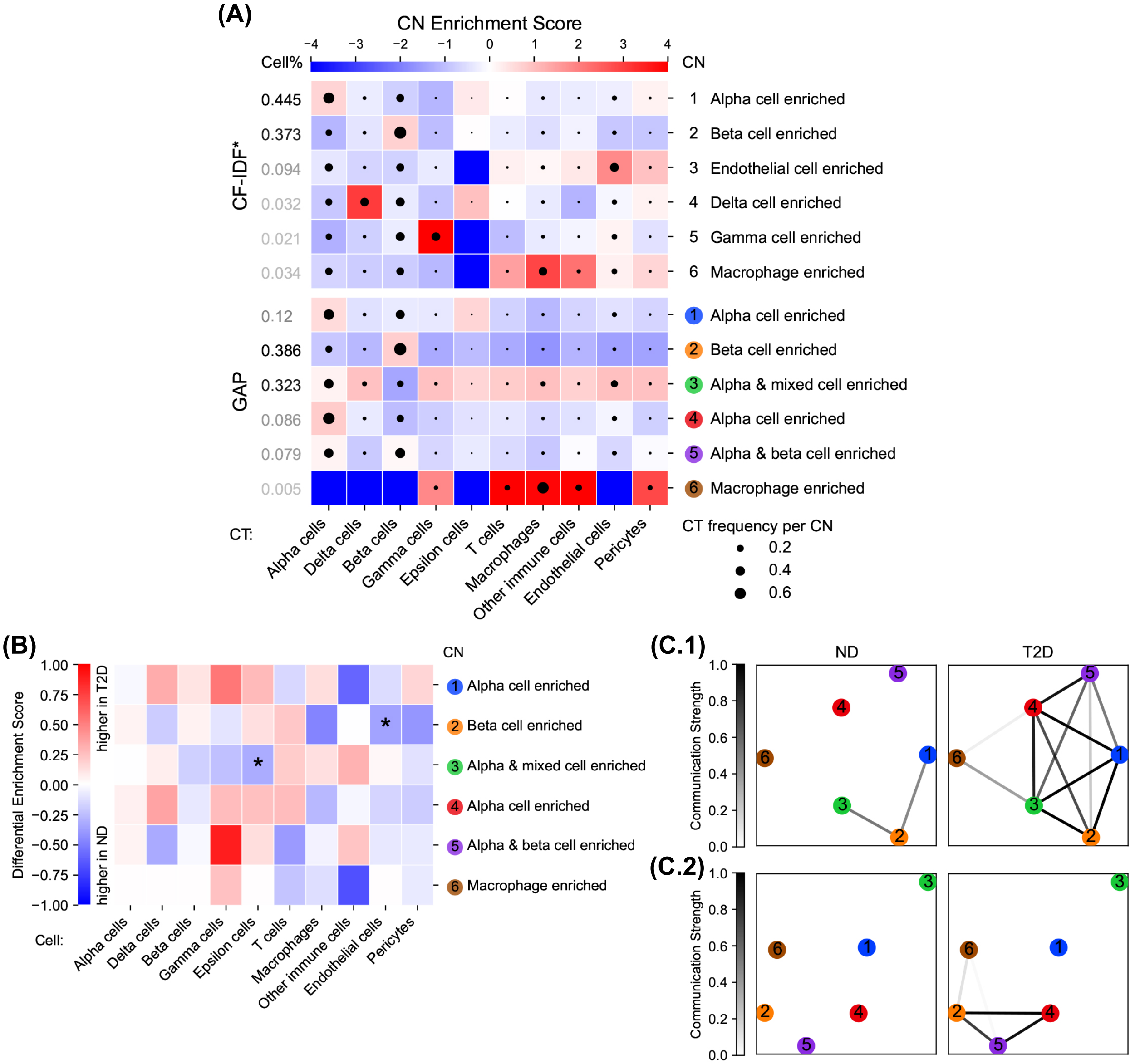

Figure N: **CN analysis results of GAP on the T2D dataset.** (A) CT Enrichment analysis. (B) Differential CT Enrichment analysis. (C.1) Inter-CN Communication Network analysis involving vascular cells (endothelial cells and pericytes). (C.2) Inter-CN Communication Network analysis involving immune cells (T cells, macrophages, and other immune cells).

Supporting figures for the HLT dataset

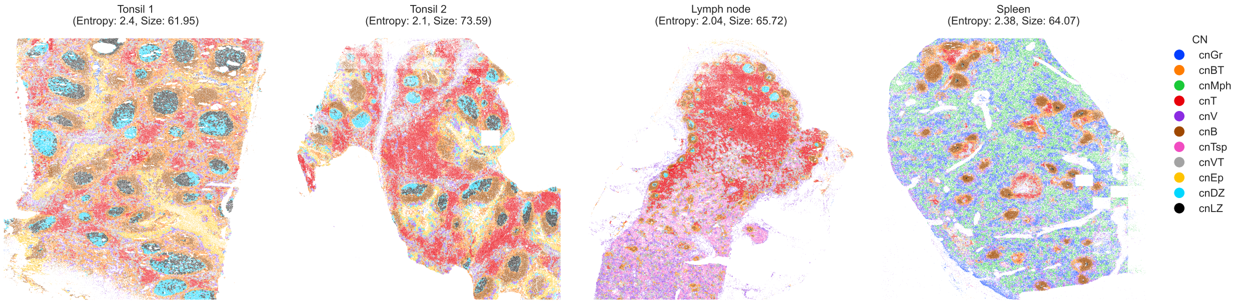

(a) CC

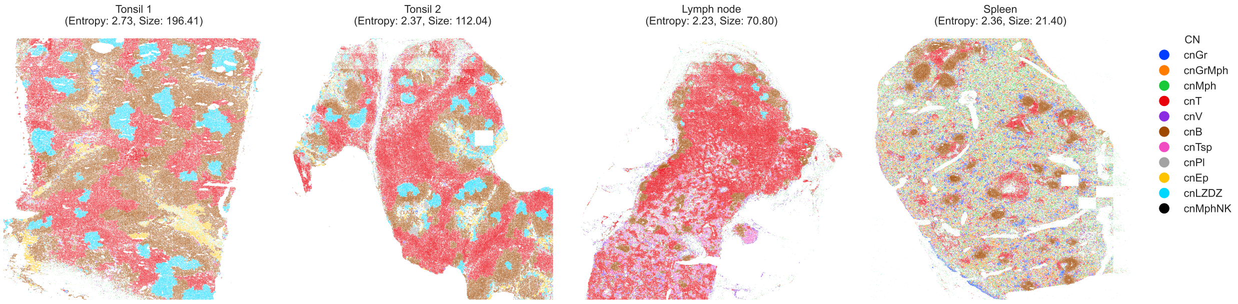

(b) CF-IDF

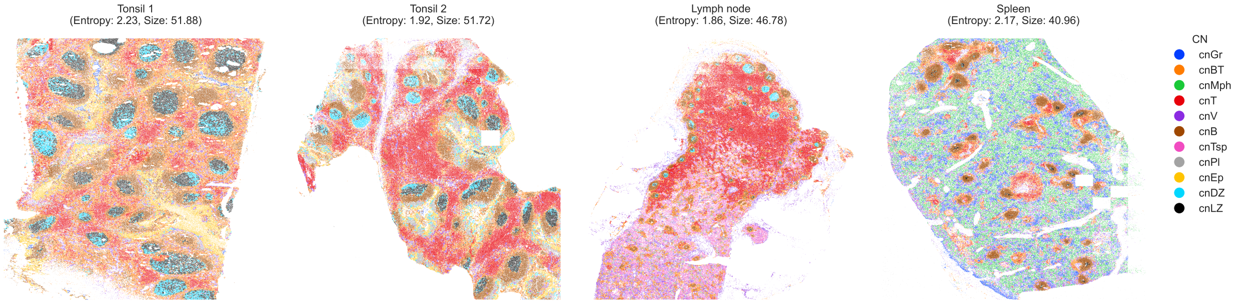

(c) CNE

Figure O: **Visualization of the identified CNs of different methods on the HLT dataset.**

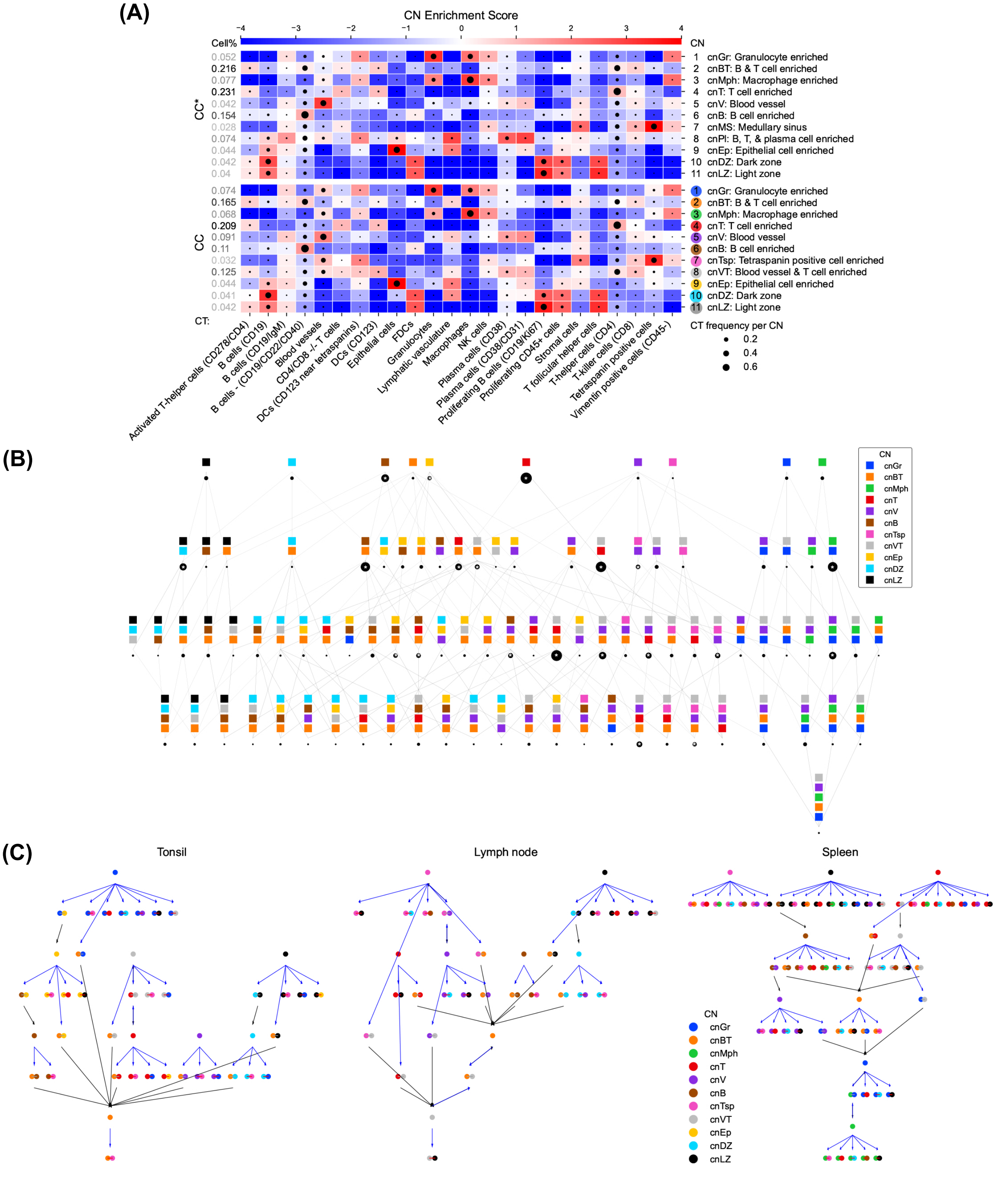

Figure P: **CN analysis results of CC on the HLT dataset.** (A) CN Combination Map analysis. (B) Assembly Rule Identification analysis.

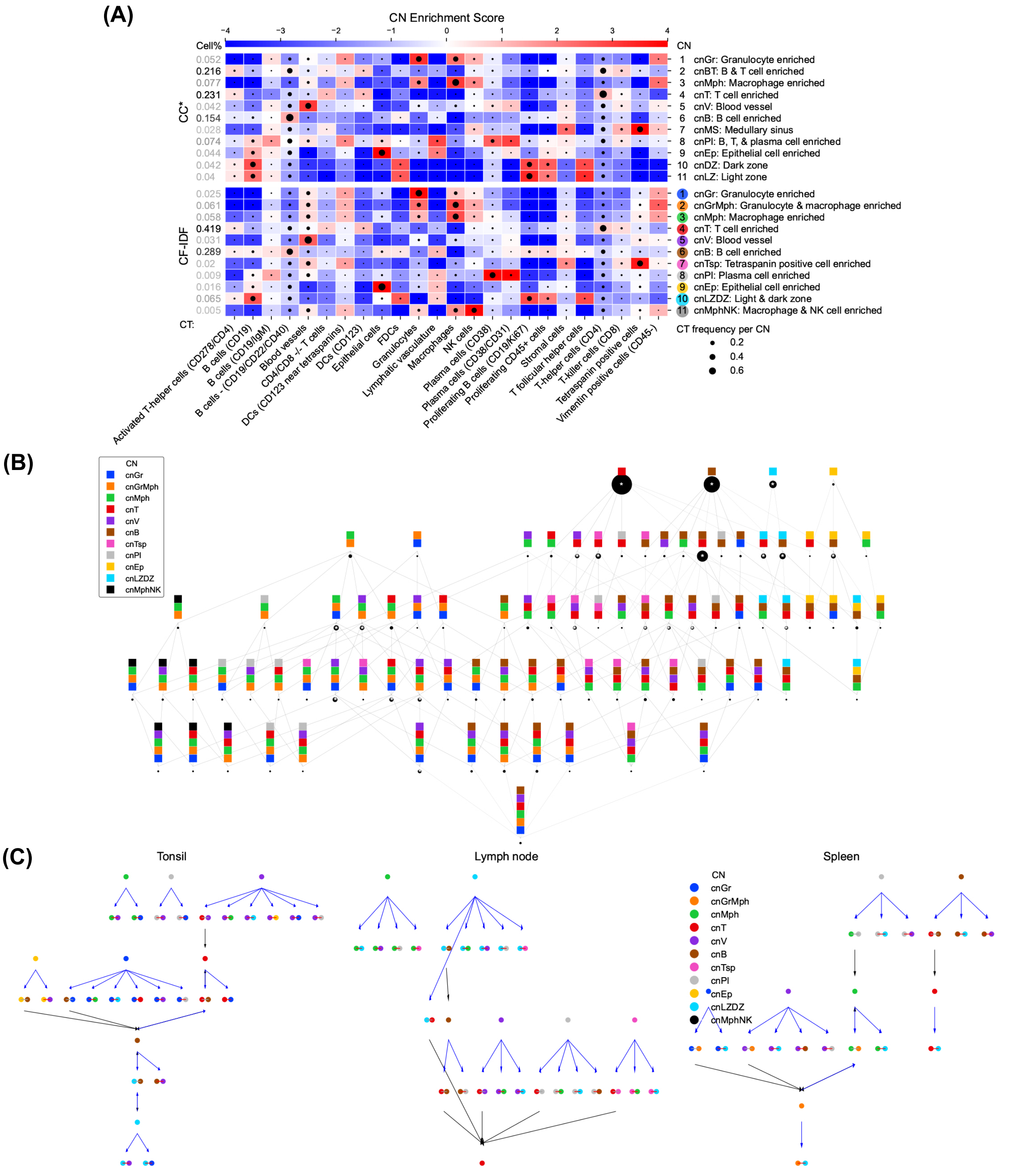

Figure Q: **CN analysis results of CF-IDF on the HLT dataset.** (A) CN Combination Map analysis. (B) Assembly Rule Identification analysis.

Sensitivity analysis of CNE on the CRC dataset

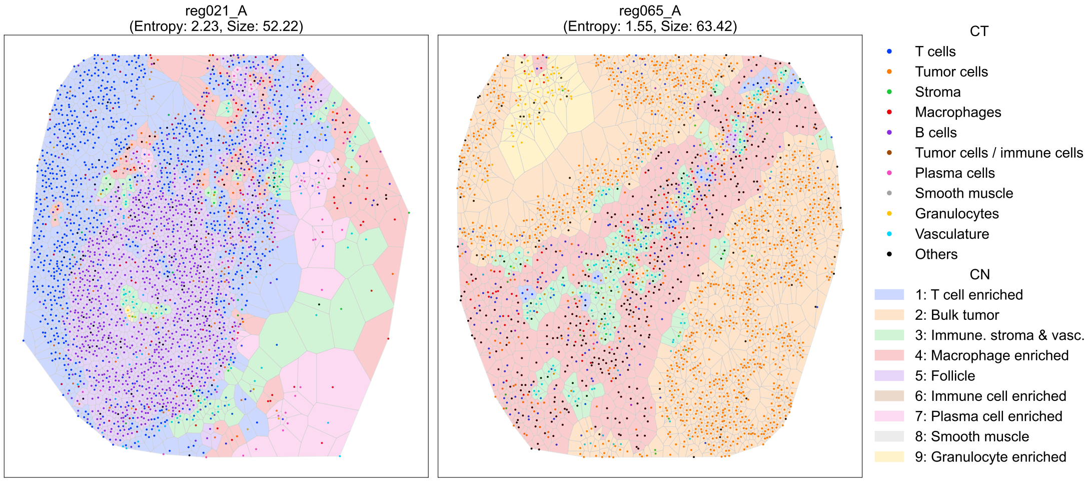

(a) CNE ($perp = 10$)

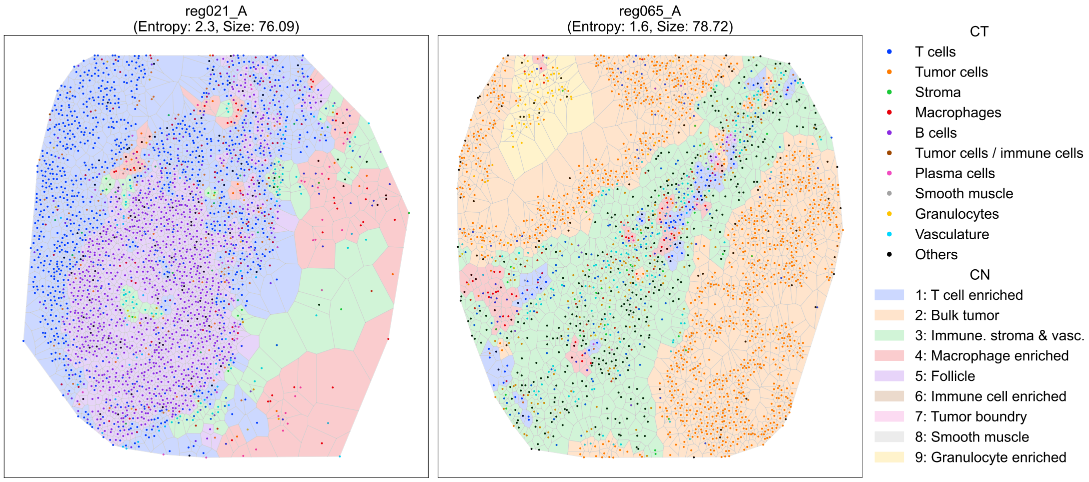

(b) CNE ($perp = 12.5$)

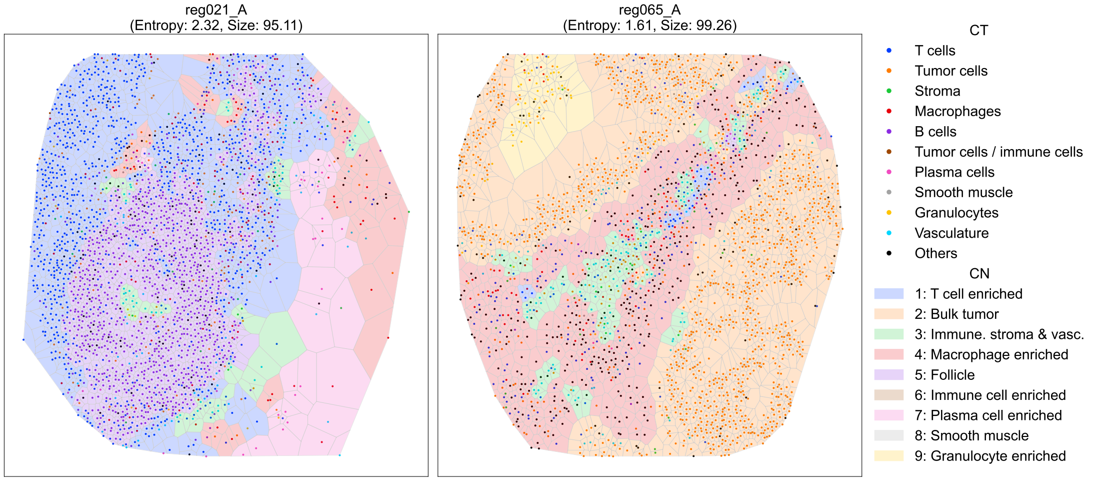

(c) CNE ($perp = 15$)

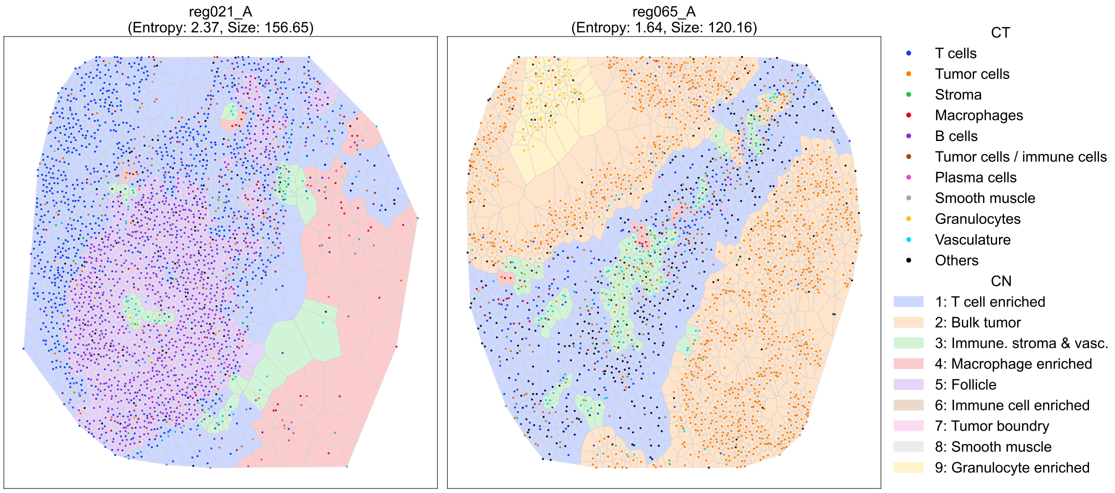

(d) CNE ($perp = 17.5$)

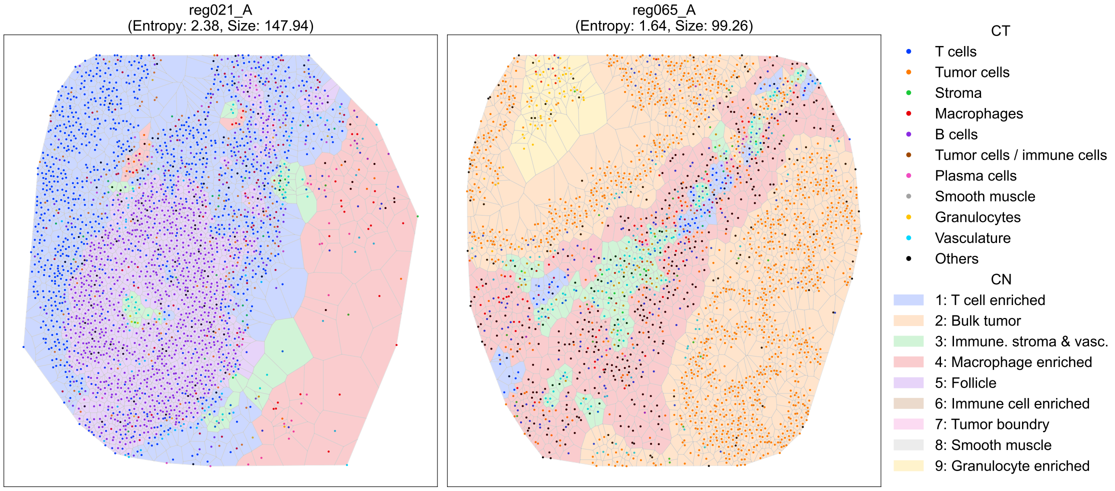

(e) CNE ($perp = 20$)

Figure R: **Voronoi diagrams showing the identified CNs of CNE on images of the CRC dataset, with hyperparameter $perp$ varying in $[10, 12.5, 15, 17.5, 20]$.** The diagrams with $perp \in \{10, 15, 20\}$ were similar, while for $perp \in \{12.5, 17.5\}$, the diagrams differed from others mainly in the diagonal region of reg065_A, which might be caused by its complex cell composition (mostly "Others" minor cell types).

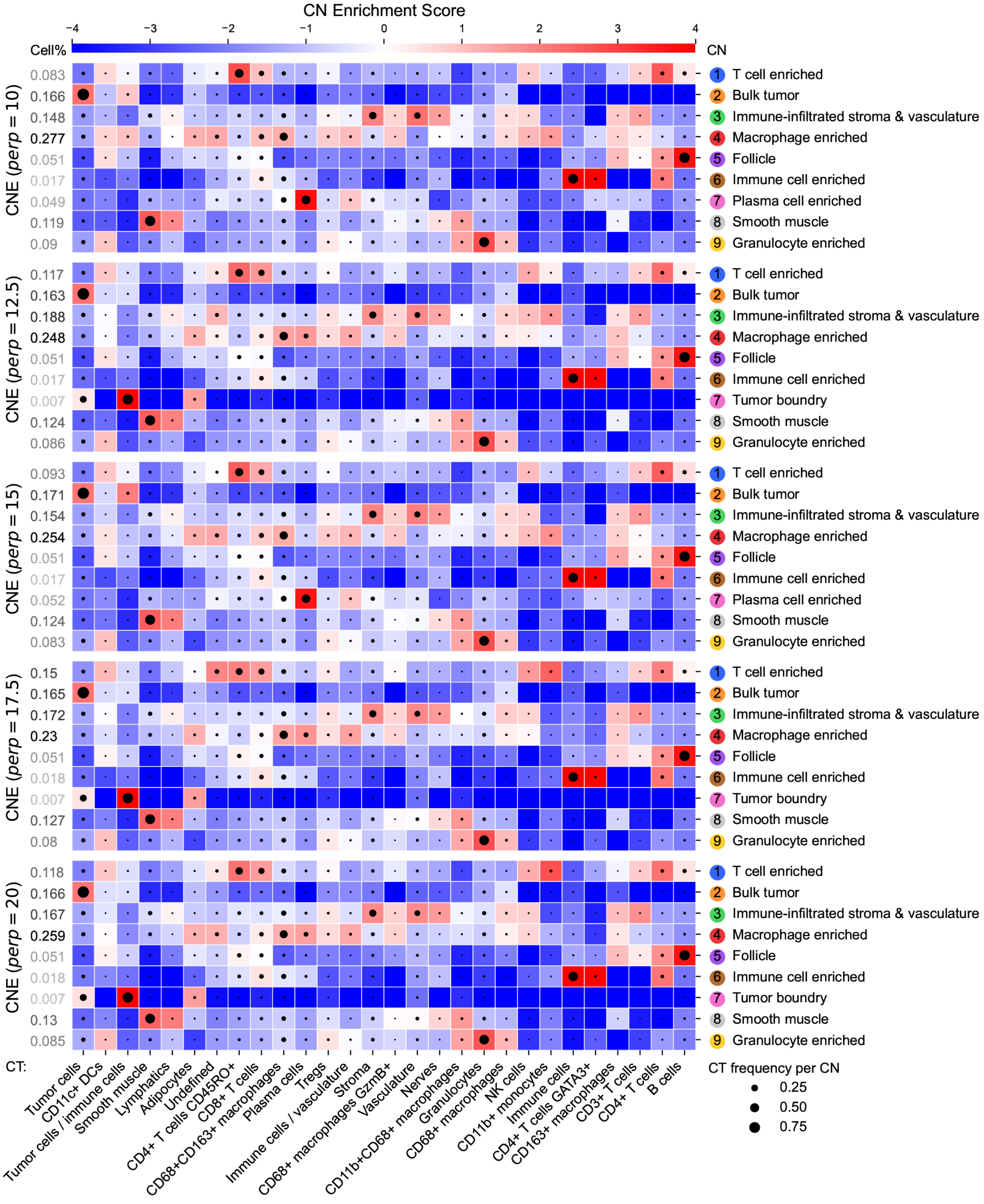

Figure S: **CT Enrichment analysis for CNE on the CRC dataset, with hyperparameter** $perp$ **varying in** $\{10, 12.5, 15, 17.5, 20\}$**.** Eight of nine CNs agreed in CT compositions across different $perp$, with CN-7 varying between plasma cell enriched CN and tumor boundary CN.

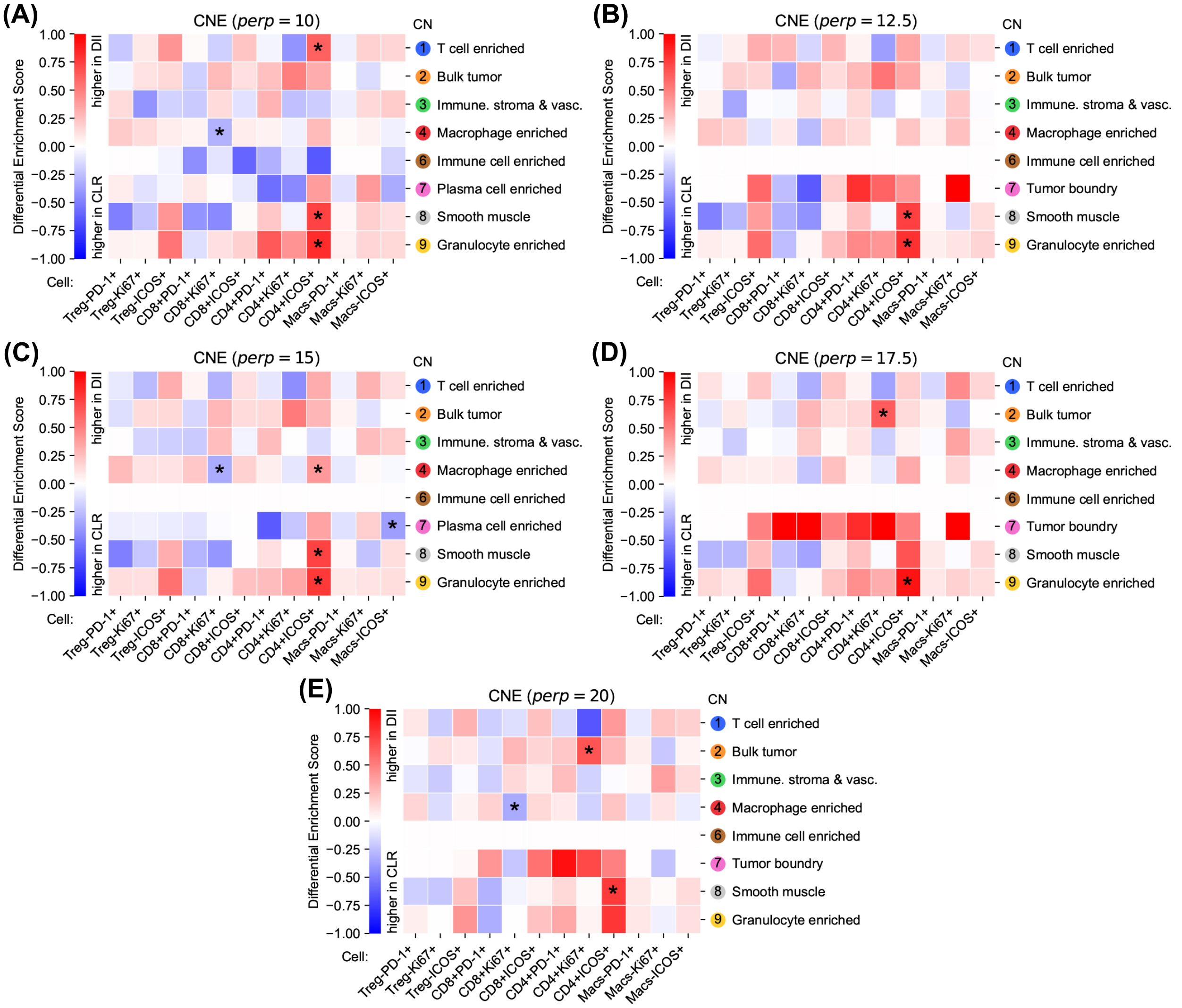

Figure T: **Differential CT Enrichment analysis for CNE on the CRC dataset, with hyperparameter *perp* varying in $\{10, 12.5, 15, 17.5, 20\}$.** Our findings of *perp*=15 could be mostly recovered by other *perp* in the range. That is, (i) Ki-67+CD8+ T cells were more enriched in the T cell enriched CN in CLR donors, Ki-67+ Treg cells were more enriched in the macrophage enriched CN in DII donors (except *perp*=20), and ICOS+ Treg cells were more enriched in the bulk tumor CN in DII donors (except *perp*=17.5), showing that immunosuppressive activity is increased in macrophage enriched and bulk tumor CNs in DII donors, while in CLR donors cytotoxic activity is increased in the T cell enriched CN; (ii) PD-1+CD4+ T cells were more enriched in the granulocyte enriched CN in DII donors, showing its potential contribution to the antitumoral response.

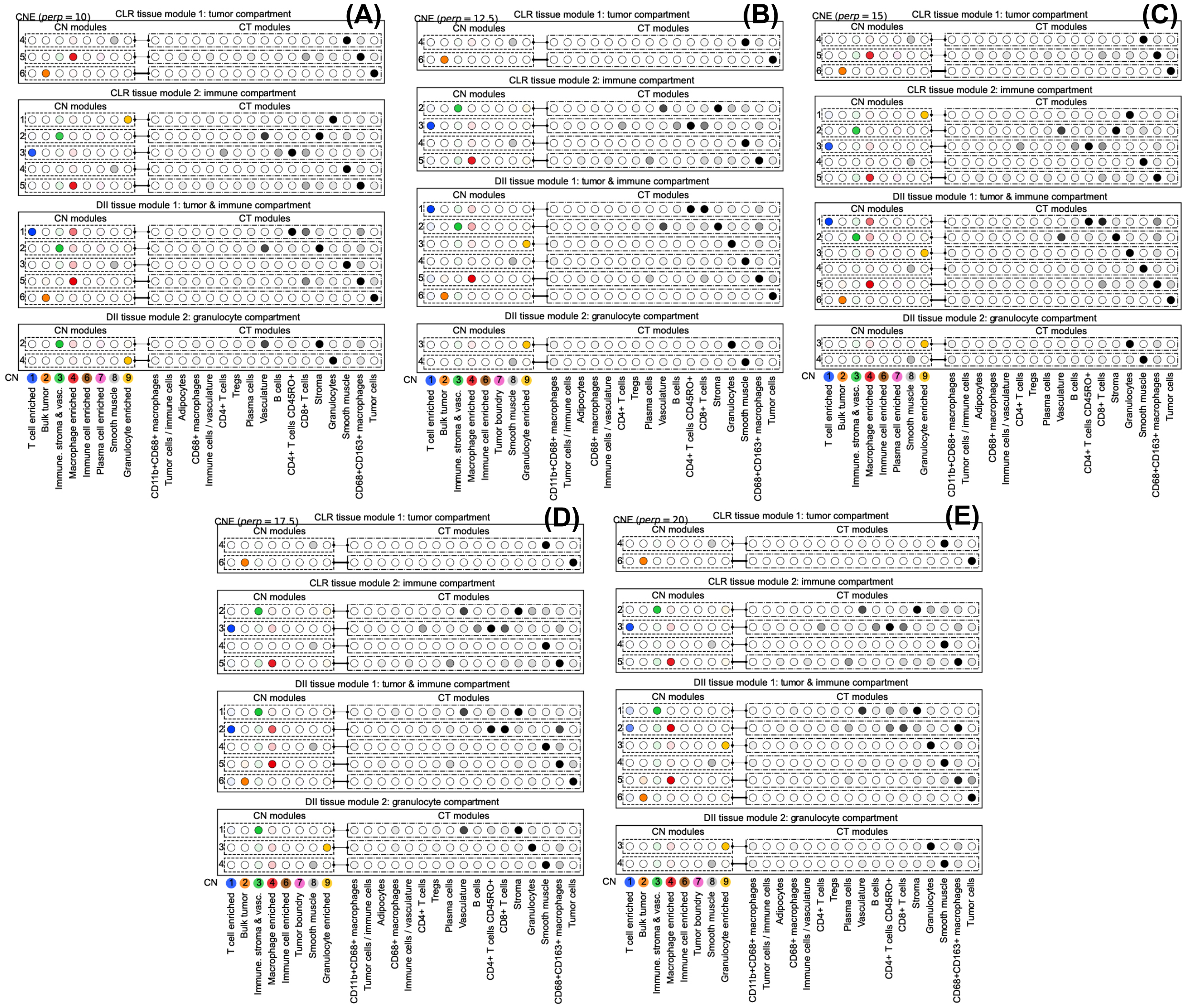

Figure U: **Tensor Decomposition analysis for CNE on the CRC dataset, with hyperparameter *perp* varying in $\{10, 12.5, 15, 17.5, 20\}$.** Our findings of *perp*=15 could be mostly recovered by other *perp* in the range. That is, (i) there was a tumor compartment and an immune compartment as tissue modules in CLR donors, and a tumor & immune compartment and a granulocyte compartment as a tissue module in DII donors; (ii) there was a CN module with high weights for T cell and macrophage enriched CNs, whose corresponding CT module had high weights for T cells and macrophages, only in DII donors, which together showed that tumors in DII donors are more correlated to the immune processes with increased coupling between T cell and macrophage enriched CNs.

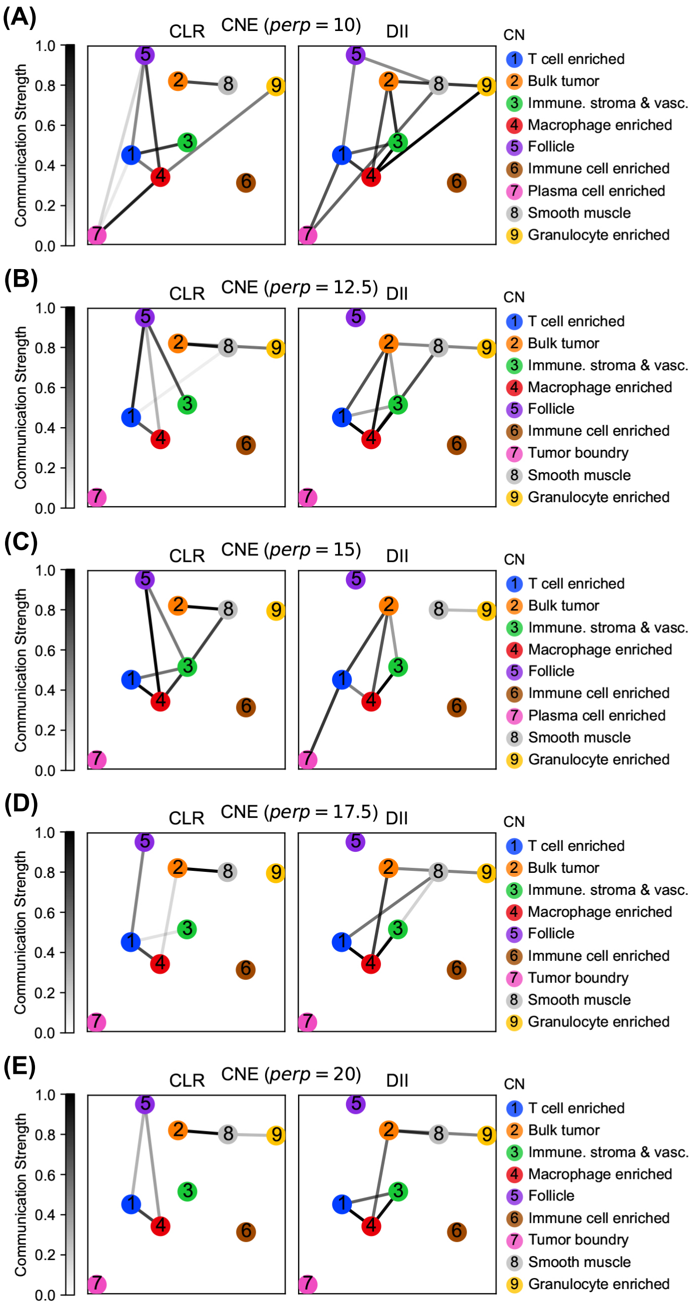

Figure V: **Inter-CN Communication Network analysis involving {PD1+, Ki-67+, ICOS+}CD8+ T cells and Ki-67+ Tregs for CNE on the CRC dataset, with hyperparameter $perp$ varying in $\{10, 12.5, 15, 17.5, 20\}$.** The follicle CN was connected to immune CNs only in CLR donors (except $perp$=12.5), and the tumor CN had stronger connection to the macrophage enriched CN in DII donors, which match our findings with hyperparameter $perp$=15. Still none of them could support the original conclusions that T cell and macrophage enriched CNs could communicate in functional T cells with the bulk tumor via the tumor boundary, and the communication between tumor boundary and bulk tumor CNs could be disrupted in DII donors.
